# Hepatitis C virus enters liver cells using the CD81 receptor complex proteins calpain-5 and CBLB

**DOI:** 10.1371/journal.ppat.1007111

**Published:** 2018-07-19

**Authors:** Janina Bruening, Lisa Lasswitz, Pia Banse, Sina Kahl, Carine Marinach, Florian W. Vondran, Lars Kaderali, Olivier Silvie, Thomas Pietschmann, Felix Meissner, Gisa Gerold

**Affiliations:** 1 Insitute for Experimental Virology, TWINCORE, Centre for Experimental and Clinical Infection Research, a joint venture between the Medical School Hannover and the Helmholtz Centre for Infection Research, Hannover, Germany; 2 Sorbonne Université, INSERM, CNRS, Centre d'Immunologie et des Maladies Infectieuses, CIMI-Paris, Paris, France; 3 Department of General, Visceral and Transplant Surgery, Hannover Medical School, Hannover, Germany; 4 Institute of Bioinformatics, University Medicine Greifswald, Greifswald, Germany; 5 Department of Experimental Systems Immunology, Max Planck Institute of Biochemistry, Martinsried, Germany; 6 Department of Clinical Microbiology, Virology, Umeå University, Umeå, Sweden; 7 Wallenberg Centre for Molecular Medicine (WCMM), Umeå University, Umeå, Sweden; University of California, San Diego, UNITED STATES

## Abstract

Hepatitis C virus (HCV) and the malaria parasite *Plasmodium* use the membrane protein CD81 to invade human liver cells. Here we mapped 33 host protein interactions of CD81 in primary human liver and hepatoma cells using high-resolution quantitative proteomics. In the CD81 protein network, we identified five proteins which are HCV entry factors or facilitators including epidermal growth factor receptor (EGFR). Notably, we discovered calpain-5 (CAPN5) and the ubiquitin ligase Casitas B-lineage lymphoma proto-oncogene B (CBLB) to form a complex with CD81 and support HCV entry. CAPN5 and CBLB were required for a post-binding and pre-replication step in the HCV life cycle. Knockout of CAPN5 and CBLB reduced susceptibility to all tested HCV genotypes, but not to other enveloped viruses such as vesicular stomatitis virus and human coronavirus. Furthermore, *Plasmodium* sporozoites relied on a distinct set of CD81 interaction partners for liver cell entry. Our findings reveal a comprehensive CD81 network in human liver cells and show that HCV and *Plasmodium* highjack selective CD81 interactions, including CAPN5 and CBLB for HCV, to invade cells.

## Author summary

CD81 is a cell membrane protein, which functions as entry factor for hepatitis C virus (HCV) and malaria sporozoites in the human liver. Currently, it remains enigmatic how CD81 guides the entry process of both pathogens and whether it functions in a similar way during liver cell invasion of HCV and malaria parasites. Here, we use high resolution quantitative proteomics to identify CD81 associated host proteins in liver cells. We found that at least 33 proteins form a complex with CD81, 23 of which were not reported as interaction partners before. We further determined that at least five CD81 interactors are HCV host factors, among them calpain-5 (CAPN5) and the ubiquitin ligase Casitas B-lineage lymphoma proto-oncogene B (CBLB). All tested HCV genotypes require CAPN5 and CBLB for full infection, but neither malaria parasites nor other tested enveloped virus rely on CAPN5 or CBLB. Our study maps the liver cell interactome of CD81 and provides new insight into the distinct cell invasion mechanisms of HCV and malaria parasites.

## Introduction

The liver is the site of initial replication of diverse parenterally transmitted pathogens. Hepatitis C virus (HCV) and the malaria parasite *Plasmodium* both enter the liver through the sinusoids and infect hepatocytes using the two host proteins CD81 and scavenger receptor class B member 1 (SCARB1) [1–5]. In particular, CD81 is essential for infection with both pathogens as mice are only susceptible to HCV when expressing human CD81 and blocking CD81 on human hepatocytes impairs *P*. *falciparum* infection [4,6]. While HCV binds to the ectodomain of CD81 and co-internalizes with CD81 into clathrin-coated vesicles [5,7], *P*. *falciparum* and the murine parasite *P*. *yoelii* do not seem to directly interact with CD81, but still require CD81 for productive uptake into hepatocytes [4,8].

HCV entry is tightly spatio-temporally controlled. After basolateral attachment of HCV to SCARB1 and subsequently CD81, the CD81-virus complex laterally translocates towards tight junctions, where the late entry factors claudin-1 (CLDN1) and occludin (OCLN) reside [9–13]. Here CD81 and CLDN1 co-internalize with the virus into endosomes [10]. The trafficking steps are thought to be coordinated by epidermal growth factor receptor (EGFR) signaling and EGFR is indeed an entry factor for HCV [14,15]. Acidification of the endosomal pH ultimately leads to fusion of the viral envelope with the limiting endosomal membrane to deliver the viral nucleocapsid into the cytoplasm [16,17]. Importantly, CD81 interaction with HCV induces conformational changes in the E1E2 surface glycoprotein heterodimer, which is a prerequisite for pH-dependent fusion [18]. After membrane fusion, the viral nucleocapsid disassembles and releases the viral genome to the cytoplasmic sites of viral genome translation and replication. This uncoating and trafficking is thought to require serum response factor binding protein 1 (SRFBP1), which is recruited to CD81 during HCV entry [19]. In sum, although CD81 fulfills multiple functions during HCV entry [20], the steady-state CD81 interaction partners required for HCV entry are largely unknown and demand elucidation.

Here we employed a combined quantitative proteomics–RNA interference (RNAi)–CRISPR/Cas9 knockout strategy to identify CD81-interacting proteins with a role in pathogen liver cell entry. Previously, we identified 26 proteins, which associate with or dissociate from CD81 during HCV entry, six of which with a role in HCV infection [19]. Here we rationalized that not only virus induced CD81 interactions, but potentially also pre-existing steady state interactions may play a role in HCV infection. Therefore, we determined high stringency CD81 protein interactions in human hepatoma cells and primary human liver cells through analysis of 120 different CD81 co-immunoprecipitations (co-IP) and high-resolution quantitative mass spectrometry. This revealed 33 CD81 interactions, ten of which were previously described. RNAi and CRISPR/Cas9 knockout follow up elucidated two proteins, the endopeptidase calpain-5 (CAPN5) and the E3 ubiquitin ligase Casitas B-lineage lymphoma proto-oncogene B (CBLB) as HCV host factors. We further confirmed a role of CAPN5 and CBLB in HCV infection of all HCV genotypes. Both proteins are expressed intracellularly and seem to affect a post-binding entry step during HCV infection. CAPN5 and CBLB are not required for *Plasmodium* uptake. Finally, we report a whole cell proteome expression dataset for human hepatoma cells and show that CAPN5 is strongly associated with CD81. Taken together, we used a combined quantitative proteomics–CRISPR/Cas9 strategy to map CD81 interactions in liver cells and identified CAPN5 and CBLB as HCV entry facilitators.

## Results

### Quantitative proteomics identifies 30 CD81 receptor interactions in hepatoma cells

**Fig 1:**
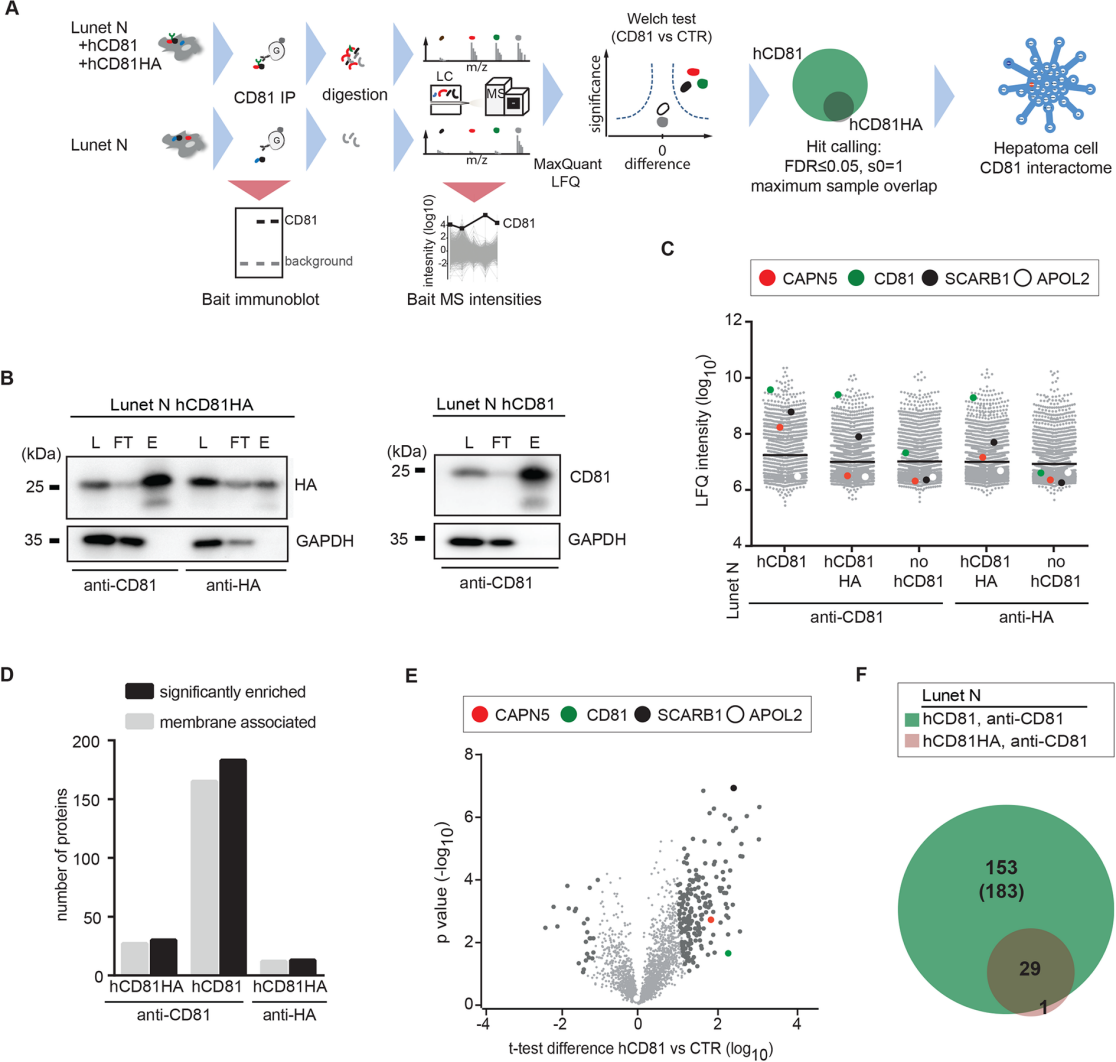
Quantitative proteomics identifies 30 CD81 receptor interactions in hepatoma cells. (A) Schematic overview of the experimental setup used to define the CD81-interactome in human hepatoma cell lines. (B) Immunoblot analysis of CD81- and HA-IPs from Lunet N hCD81HA and Lunet N hCD81 cells using anti-CD81 or anti-HA antibodies, as indicated. GAPDH served as loading control. L = lysate, FT = flow through, E = eluate. Representative of 4 biological replicates. (C) LFQ intensities of proteins in CD81- and HA-IPs from the indicated cell line. CD81 (green) and SCARB1 (black) served as positive and APOL2 (white) as negative control. CAPN5 (red) was discovered as CD81 interactor in hepatoma cells. Median of 4 biological replicates. (D) Number of proteins significantly enriched in the indicated co-IPs and membrane associated fraction. (E) Volcano plot visualizing two-sample t-test comparing LFQ intensities of proteins found in CD81-IPs from Lunet N hCD81 and Lunet N. For each protein the t-test difference (log_10_) of CD81 versus control co-IP of 4 biological replicates is plotted against the p value (-log_10_). FDR = 0.01; s0 = 2. Proteins significantly enriched are highlighted in dark grey. CD81 (green), SCARB1 (black) APOL2 (white) and CAPN5 (red) are highlighted. (F) Overlap of significantly enriched proteins found in anti-CD81co-IPs from Lunet N hCD81 and Lunet N hCD81HA. See also S1 Fig, S2 Fig, S1 Table and S2 Table.

Pathogens engage host cell protein networks to gain access into replication competent intracellular compartments [21–23]. Mass spectrometry based-proteomics has matured into a powerful technology to comprehensively analyze protein-protein interactions (PPIs) from cell culture and primary cell material [22–24]. Here we set out to apply label-free quantitative (LFQ) proteomics to map the interaction network of the HCV and *Plasmodium* entry factor CD81 in human liver cells (Fig 1A). Therefore, we used human hepatoma cell lines (Lunet N) lacking endogenous CD81 and expressed wildtype or C-terminally hemagglutinin- (HA) tagged CD81 in these cells (S1A Fig). This allowed us to use antibodies against endogenous or tag epitopes to pull out different versions of the bait. We confirmed expression of the respective CD81 protein by immunoblot, immunofluorescence microscopy and flow cytometry. Wildtype and HA-tagged CD81 were expressed at the cell surface and in intracellular compartments comparable to endogenous CD81 in Huh-7.5 human hepatoma cells (S1B–S1D Fig). Lunet N cells lacking detectable CD81 expression served as negative control in all infection and proteomics experiments [25]. Expectedly, Lunet N cells lacking CD81 were refractory to infection with HCV and with lentiviruses pseudotyped with HCV glycoproteins, which rely on HCV receptor interaction during entry [26,27]. In contrast expression of hCD81 or hCD81HA in Lunet N cells rendered cells HCV susceptible (S1E and S1F Fig).

Next we determined which co-IP methods preserved known CD81 interactions. Comparison of various detergents revealed that Brij-58 preserved most known CD81 interactions such as the interaction with SCARB1 while excluding proteins not expected to interact with CD81 such as GAPDH and apolipoprotein L2 (APOL2) (Fig 1B and 1C and S2A–S2C Fig). Moreover, we evaluated the use of the homobifunctional amine-reactive cell membrane impermeable crosslinker (bis-sulfosuccinimidyl suberate, BS3). BS3 crosslinking allowed pull out of CD81 and the control prey SCARB1. Nonetheless, BS3 crosslinking reduced CD81 and SCARB1 enrichment by one or two orders of magnitude, respectively (intensity (CD81): 10^10^, intensity (CD81, BS3): 10^9^, intensity (SCARB1): 10^9^, intensity (SCARB1, BS3): 10^7^) (S2D Fig). This reduced capture may result from crosslinking of large protein aggregates, which are lost during preclearing of lysates or from masking of antibody epitopes. We therefore decided to primarily analyze CD81 networks from Brij-58 lysed, non-crosslinked cells.

To map the CD81 PPI network in human hepatoma cells, we performed a total of 64 CD81 co-IPs in quadruplicates from Lunet cells lacking or expressing CD81. We further compared enrichment data from endogenous CD81 bait and HA-tagged CD81 bait. On average our mass spectrometric analysis identified 2600 proteins with a false discovery rate (FDR) of 1% on the peptide and protein level (S1 Table).

The CD81 bait was strongly enriched (2 to 3 orders of magnitude) in all CD81 IPs but not in control IPs (Fig 1C). As expected positive (SCARB1) and negative controls (APOL2) showed high and low enrichment, respectively. Among the strongly enriched, previously unknown CD81 interactors, we identified the endopeptidase CAPN5.

In total, we identified 183 significant proteins in co-immunoprecipitations from hCD81 expressing hepatoma cells using an antibody targeting the CD81 LEL (Fig 1D and 1E). The majority (approximately 90%) of detected CD81 interacting proteins are membrane associated as determined by GO enrichment analysis (Fig 1D). Interestingly, we detected only 30 CD81 interactors (FDR = 0.07, s0 = 1) in hCD81HA expressing cells using the same CD81 antibody and despite seemingly similar subcellular localization of hCD81HA (S1D Fig). Possibly, tagging the CD81 cytoplasmic tail weakens a subset of PPIs. Moreover, we observed even weaker protein enrichment when using an anti-HA antibody, indicating that pulling on the ectodomain of CD81 may preserve more interactions than pulling on the C-terminus. As hCD81HA expressing cells showed similar susceptibility to HCV as hCD81 expressing cells (S1E and S1F Fig), we argued that HCV infection relevant interactions should be preserved for hCD81HA. Of the 30 detected hCD81HA interactions, 29 overlapped with the hCD81 interactome (Fig 1F and Table 1).

**Table 1:** Proteins in the human liver cell CD81 complex.

			log_10_ intensity differences				
			Lunet N hCD81	Lunet N CD81HA		PHH D1	PHH D2
Protein names	Gene names	UniProt ID	anti-CD81, ctrl		anti-HA, ctrl	anti-CD81, IgG	
CD166 antigen	ALCAM	Q13740	2.581	2.203	0.859	0.898	0.776
Annexin A11	ANXA11	P50995	2.535	2.080	0.596	1.204	0.368
Apolipoprotein A-I;Proapolipoprotein A-I;Truncated apolipoprotein A-I	APOA1	P02647	1.590	1.464	0.317	0.182	1.295
Apolipoprotein E	APOE	P02649	1.678	1.841	1.600	0.139	1.711
Sodium/potassium-transporting ATPase subunit alpha-1	ATP1A1	P05023	1.654	1.440	0.617	0.432	0.703
Uncharacterized protein C2orf72	C2orf72	A6NCS6	2.038	2.322	1.285	1.777	0.945
Calpain-5	CAPN5	O15484	1.905	0.840	0.149	1.986	2.049
CD151 antigen	CD151	P48509	1.905	1.744	0.050	1.458	2.319
Membrane cofactor protein	CD46	P15529	1.799	1.037	1.077	0.701	1.125
CD81 antigen	CD81	P60033	2.244	1.964	2.778	2.502	1.670
Epidermal growth factor receptor	EGFR^+#^	P00533	1.923	1.680	1.476	1.654	-0.211
Ectonucleotide pyrophosphatase/phosphodiesterase family member 1;Alkaline phosphodiesterase I;Nucleotide pyrophosphatase	ENPP1	P22413	1.610	0.630	0.391	1.694	1.734
Junctional adhesion molecule A	F11R	Q9Y624	1.693	1.558	1.076	1.488	0.658
Guanine nucleotide-binding protein subunit alpha-11	GNA11^#^	P29992	2.123	1.856	0.978	1.905	1.523
Guanine nucleotide-binding protein subunit alpha-13	GNA13	Q14344	2.365	1.770	0.722	1.845	1.371
Guanine nucleotide-binding protein G(i) subunit alpha-1	GNAI1	P63096	1.421	0.779	0.179	1.475	2.263
Guanine nucleotide-binding protein G(i) subunit alpha-2	GNAI2^#^	P04899	1.729	1.342	1.105	1.475	2.263
Guanine nucleotide-binding protein G(k) subunit alpha	GNAI3	P08754	2.302	1.466	1.135	1.001	1.204
Guanine nucleotide-binding protein G(s) subunit alpha isoforms short;Guanine nucleotide-binding protein G(s) subunit alpha isoforms XLas	GNAS	P63092, Q5JWF2	2.444	1.597	0.988	1.867	0.645
Guanine nucleotide-binding protein G(I)/G(S)/G(T) subunit beta-1	GNB1^#^	P62873	1.752	1.166	1.642	1.462	1.751
Guanine nucleotide-binding protein G(I)/G(S)/G(T) subunit beta-2	GNB2^#^	P62879	1.510	1.319	1.282	1.761	0.782
Integrin alpha-1	ITGA1	P56199	2.334	1.294	0.210	1.782	1.704
Integrin alpha-6;Integrin alpha-6 heavy chain;Integrin alpha-6 light chain;Processed integrin alpha-6	ITGA6	P23229	3.054	1.942	0.752	0.964	0.033
Integrin beta-1	ITGB1^#^	P05556	2.596	2.228	1.053	1.818	2.413
Prostaglandin F2 receptor negative regulator	PTGFRN	Q9P2B2	2.713	1.453	-0.491	2.397	2.615
Poliovirus receptor	PVR	P15151	1.790	1.576	-0.303	0.962	0.842
Rho-related GTP-binding protein RhoB	RHOB	P62745	1.990	0.735	0.430	0.845	1.979
Scavenger receptor class B member 1	SCARB1^+^	Q8WTV0	2.410	1.536	1.429	1.874	1.617
Zinc transporter ZIP14	SLC39A14	Q15043	1.691	1.565	0.022	1.403	0.492
4F2 cell-surface antigen heavy chain	SLC3A2	P08195	2.229	1.621	0.776	1.538	0.789
Serotransferrin	TF	P02787	2.479	1.810	1.296	0.715	0.789
Transferrin receptor protein 1;Transferrin receptor protein 1, serum form	TFRC^+#^	P02786	2.570	2.030	1.462	1.488	1.198
Transmembrane protein 2	TMEM2^#^	Q9UHN6	3.080	2.852	1.896	1.738	0.748
Vesicle-associated membrane protein-associated protein A	VAPA	Q9P0L0	1.797	0.408	0.777	0.778	1.332
E3 ubiquitin-protein ligase CBL	CBL*^+^	P22681	n.d.	n.d.	n.d.	n.d.	n.d.
E3 ubiquitin-protein ligase CBL-B	CBLB*	Q13191	n.d.	n.d.	n.d.	n.d.	n.d.
Guanine nucleotide-binding protein G(I)/G(S)/G(O) subunit gamma-2	GNG2*	P59768	n.d.	n.d.	n.d.	n.d.	n.d.
Guanine nucleotide-binding protein G(I)/G(S)/G(O) subunit gamma-4	GNG4*	P50150	n.d.	n.d.	n.d.	n.d.	n.d.
Guanine nucleotide-binding protein G(I)/G(S)/G(O) subunit gamma-5	GNG5*	P63218	n.d.	n.d.	n.d.	n.d.	n.d.
Guanine nucleotide-binding protein G(I)/G(S)/G(O) subunit gamma-7	GNG7*	O60262	n.d.	n.d.	n.d.	n.d.	n.d.
Growth factor receptor-bound protein 2	GRB2*^+^	P62993	n.d.	n.d.	n.d.	n.d.	n.d.
Integrin alpha-5;Integrin alpha-5 heavy chain;Integrin alpha-5 light chain	ITGA5*	P08648	2.213	1.586	0.293	0.327	0.590
SHC-transforming protein 1	SHC1*^+^	P29353	n.d.	n.d.	n.d.	n.d.	n.d.

Proteins identified as CD81 interaction partners by affinity enrichment mass spectrometry and in silico prediction (STRING, labeled with *) are listed. Previously reported HCV entry factors and facilitators are labeled with ^+^. Interaction partners detected in the presence of competing soluble CD81 LEL are labeled with ^#^. The enrichment of each protein in the indicated co-IP is listed as median value of four biological replicates. Shaded in grey are proteins significant in hepatoma cells / ≥4-fold enriched in PHH. n.d. not detected.

CD81 is a tetraspanin consisting of four transmembrane domains, one small and one large extracellular loop (LEL) and short cytosolic domains. The LEL binds directly to the E2 glycoprotein ectodomain on HCV particles. As it was unclear which CD81 domains mediate host PPIs, we measured an additional CD81 interactome from Lunet hCD81HA cells in the presence of a molar excess of soluble CD81-LEL. Competition for the LEL binding sites led to a strong reduction of CD81 PPIs. We found only eight of the 29 PPIs detected in the absence of LEL (labeled with # in Table 1 and S2D, S2F and S2G Fig). This underlines the important function of the CD81 LEL not only in virion binding, but also in molecular interactions with host proteins.

Of the 29 high confidence and potentially HCV entry relevant CD81 interacting proteins, seven were previously detected CD81 interactors, namely SCARB1, the integrins ITGA6 and ITGB1, the G proteins GNA11 and GNAI3, the tetraspanin and postulated papilloma virus entry factor CD151 and apolipoprotein E (ApoE) [15]. Moreover, four proteins were reported HCV entry facilitators namely SCARB1, epidermal growth factor receptor (EGFR), transferrin receptor protein 1 (TFRC) and the ubiquitin ligase CBL (labeled with + in Table 1) [1,14,28,29]. Taken together we discovered 29 CD81 interactors in human hepatoma cells, 17 of which are novel CD81 interactors without a previously reported function in CD81 binding or HCV cell invasion (see S1 Table for full dataset).

### Stratification of 33 CD81 receptor interactions in primary human hepatocytes

**Fig 2:**
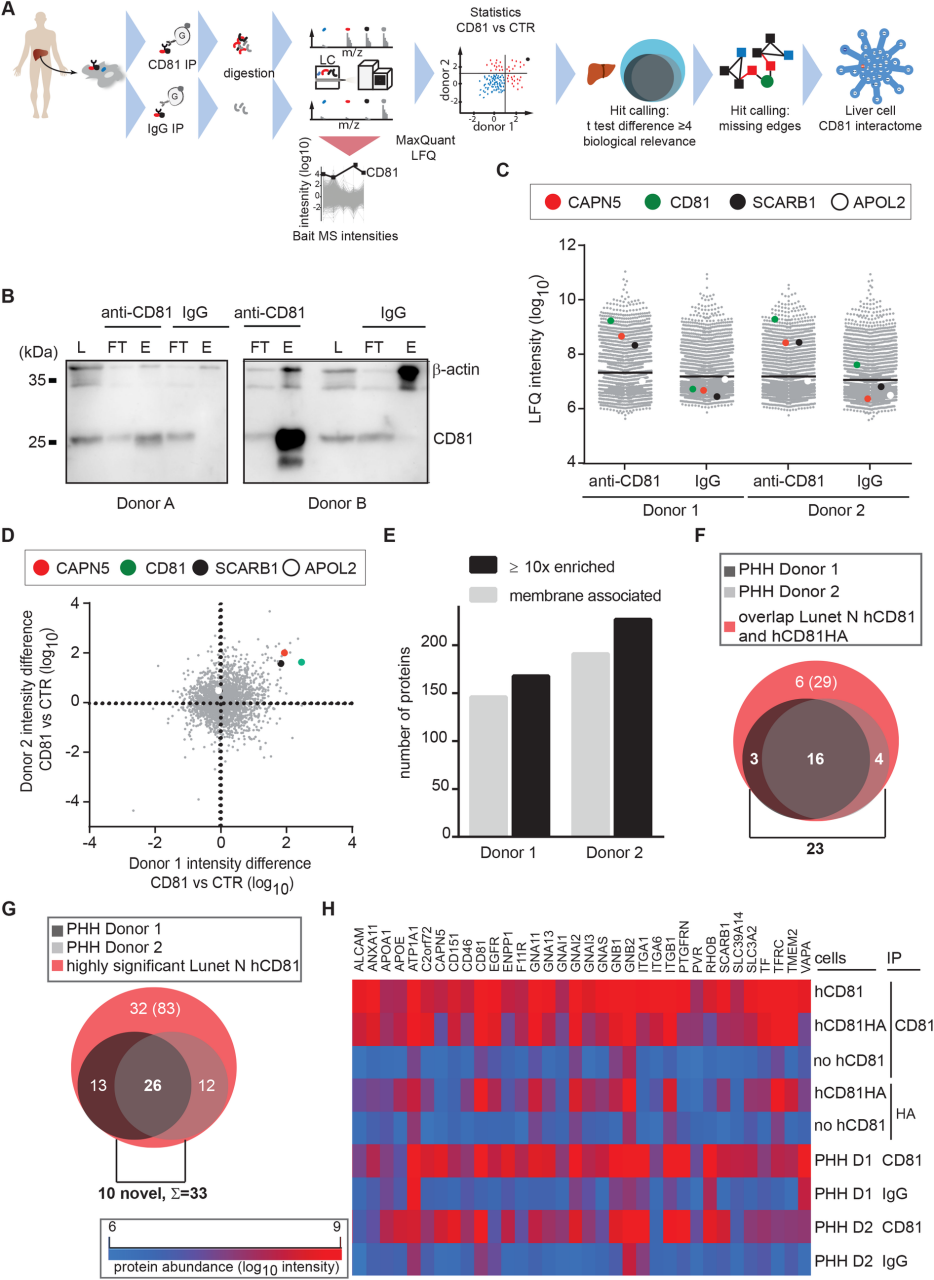
Stratification of 33 CD81 receptor interactions in primary human hepatocytes. **(**A) Schematic overview of the experimental setup used to define the CD81-interactome in primary human hepatocytes (PHH). (B) Immunoblot analysis of CD81- and IgG-IPs from PHH of two donors using an anti-CD81 antibody. Actin served as loading control. L = lysate, FT = flow through, E = eluate. (C) LFQ intensities of proteins in CD81- or IgG-IPs from PHH of two independent donors. CD81 (green) and SCARB1 (black) served as positive and APOL2 (white) as negative control. CAPN5 (red) was discovered as CD81 interactor in PHH. (D) Scatter plot comparing intensity differences of proteins found in CD81- versus IgG-IPs in two donors of PHH. CD81 (green), SCARB1 (black), APOL2 (white) and CAPN5 (red) are highlighted. (E) Number of proteins found ≥ 10-fold enriched in the indicated co-IPs and membrane associated protein fraction. (F) 23 proteins found at least 4-fold enriched in CD81-IPs from PHH donor 1 and 2 and significantly enriched in co-IPs from Lunet N hCD81 and Lunet N hCD81HA. (G) 26 proteins identified in CD81-IPs from PHH donor 1 and 2 and in Lunet N hCD81 cells with high stringency (FDR < 0.004). Among the 26 proteins, 16 overlapped with the analysis in (F), resulting in a total of 33 stringent CD81 interactors in PHH and hepatoma cells. (H) Heat map showing protein abundance as median intensity (log_10_) for the 33 hits and the CD81 bait in indicated co-IP samples. Red and blue colors indicate high or low intensity difference, respectively. See also S3 Table.

**Fig 3:**
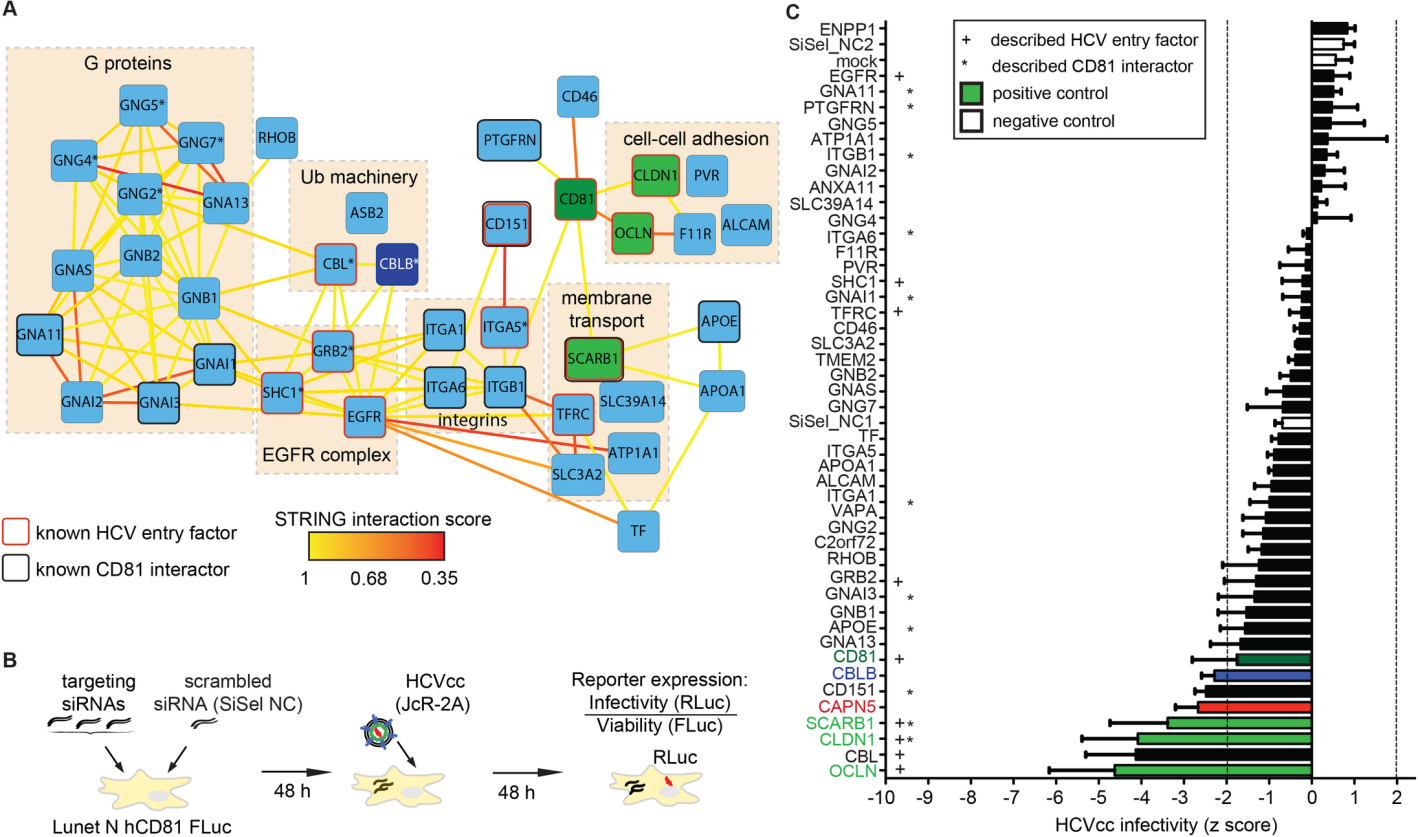
A subset of CD81 interacting proteins is required for full HCV infectivity. **(**A) Functional map of host factors interacting with the HCV receptor CD81. Functional clusters (boxes) and previously reported interactions (bold lines) of the identified CD81 binding partners and the HCV entry factors OCLN and CLDN1 are depicted. Yellow lines between genes of different clusters indicate high-confidence (>0.9) STRING interactions. Lower confidence (>0.35) STRING interactions are shown as red lines. Nine highest scoring additional nodes (indicated by asterisk) were included for follow up analysis. The full set of identified proteins is depicted in S3A Fig. (B) Experimental setup of the siRNA screen used to identify CD81 interactors important for HCV infection. (C) Human hepatoma cells were transfected with a pool of three siRNAs targeting the 42 CD81-interactors or with a scrambled non-targeting control (SiSel NC), followed by infection with a HCV luciferase reporter virus (JcR-2A). Infectivity was measured 48 hpi as luciferase activity and normalized for cell viability and plate effects. Knock down of four CD81-interactors significantly decreased HCV infection (p≤ 0.05; abs (z score) ≥ 2). Data from 3 biological replicates shown as mean +SEM. See also S3 Fig.

To unravel, whether the identified CD81 protein interaction network in hepatoma cells reflected the PPIs in primary human hepatocytes, we developed a second LFQ affinity enrichment mass spectrometry (AE-MS) pipeline (Fig 2A). Here due to lack of control cells negative for CD81 expression, we performed isotype control co-IPs to determine nonspecific background binding of proteins to the IP resin (Fig 2B). We analyzed primary human hepatocytes from five donors. Samples from two donors passed our quality criteria namely strong CD81 bait and SCARB1 positive control enrichment (>10-fold). An irrelevant protein (APOL2) showed similarly low abundance in CD81 specific and control IPs (Fig 2C and 2D). More than 150 proteins were enriched more than 10-fold over controls in both donors. Of these proteins roughly 80% are annotated as membrane associated (Fig 2E and S3 Table). Twenty-three CD81 interactors found in primary human hepatocytes overlapped with the set of 29 hepatoma cell interactors (Fig 2F and Table 1). As prey protein enrichment was limited in the co-IPs from HA-tagged CD81 expressing cells, we performed a second overlap analysis exclusively with the primary hepatocyte hits (>4-fold enrichment) and highly significant hepatoma cell hits (FDR = 0.004, s0 = 2) from cells with untagged CD81. Through this second analysis, we detected an overlap of 26 proteins including ten ‘novel’ proteins (Fig 2G) not found in the first overlap analysis (Fig 2F). Of note, these ten hits were also enriched in anti-CD81 co-IPs from CD81HA expressing cells, but not to an extent to fulfill our very stringent hit inclusion criteria of roughly 30-fold enrichment (Table 1). The 10 novel hits together with the 23 hits from the first overlap analysis resulted in 33 high confidence CD81 interactors in primary hepatocytes and hepatoma cells (Fig 2G and Table 1). Fig 2H summarizes the protein abundance of the CD81 bait protein and the 33 CD81 interactors in co-IPs from hepatoma cells and primary human hepatocytes. Lastly, we performed an integrated network analysis based on STRING and DAVID data for the 33 conserved CD81 binding partners and showed that 28 proteins are highly interconnected through previously reported interactions (Fig 3A and Table 1). From this network, we included the closest nine nodes in our hit list for follow up, resulting in a final hit list of 42 proteins. This ensured that putative network proteins poorly amenable to MS identification were still included in subsequent infection assays (in silico interactors labeled with * in Table 1). Of the final 42 CD81 network molecules defined in this study, ten proteins were previously recognized CD81 interactors including SCARB1, the integrins ITGA1, ITGA6, ITGB1, the G proteins GNA11, GNAI1, GNAI3, CD151, ApoE and prostaglandin F2 receptor negative regulator (PTGFRN) [15]. Six of the CD81 liver cell interactors found here were previously reported HCV entry facilitators as described above (labeled with ^+^ in Table 1). In addition, the LFQ approach and extensive comparison of hepatoma and primary cell datasets elucidated 27 CD81 interactors without a previously reported role in HCV entry or CD81 binding. In summary, we defined a stringent set of 42 CD81 interactors in human liver cells (S3A Fig, Table 1).

### A subset of CD81 interacting proteins is required for full HCV infectivity

CD81 is an essential factor for HCV and *Plasmodium* sporozoite entry into liver cells [4,5]. As CD81 mediates its molecular functions through PPIs, we hypothesized that a subset of CD81 interactions is required by both pathogens to productively infect liver cells. Within the 42 CD81 network proteins, we indeed found proteins previously reported as HCV entry facilitators such as integrins, apolipoproteins and EGFR complex molecules (Fig 3A).

To unravel yet unknown CD81 interacting proteins acting as pathogen entry facilitators we investigated human hepatoma cell susceptibility to HCV after silencing each factor (Fig 3B). Expectedly, silencing the four established HCV entry factors CD81, SCARB1, CLDN1 and OCLN reduced susceptibility to HCV with mean z-scores of -1.8, -3.4, -4.1 and -4.6, respectively (‘positive controls’ in Fig 3C). The CD81 silencing effect was limited since we used Lunet N hCD81 cells, which overexpress hCD81. Five CD81 network proteins, namely the E3 ubiquitin ligases CBLB and CBL, the endopeptidase CAPN5, SCARB1 and CD151, reduced the susceptibility to HCV upon siRNA silencing with mean z-scores of -2.3, -4.1, -2.7, -3.4 and -2.4, respectively (Fig 3C). Among these putative HCV entry facilitators were the established HCV entry factor SCARB1, the entry facilitator CBL and the previously described CD81 interactor CD151 [15,29]. Moreover, we found two novel putative entry facilitators. These are CBLB and CAPN5. While we discovered CAPN5 as CD81 interaction partner in hepatoma cells and primary human hepatocytes (Figs 1 and 2), we included CBLB as a hit from the STRING network analysis (Fig 3A). Silencing efficiency was not assessed in this RNAi screen. Hence we cannot rule out that additional CD81 complex proteins facilitate HCV infection. The data underline that CD81 interacting proteins function in HCV entry. The discovery of CBL and CBLB as HCV host factors are in line with findings showing that ubiquitination events are required in endocytosis and entry of viruses of diverse families including influenza virus and adenoviruses [30,31].

### *Plasmodium* Sporozoite hepatoma cell entry is associated with a distinct usage of the CD81 protein interaction network

**Fig 4:**
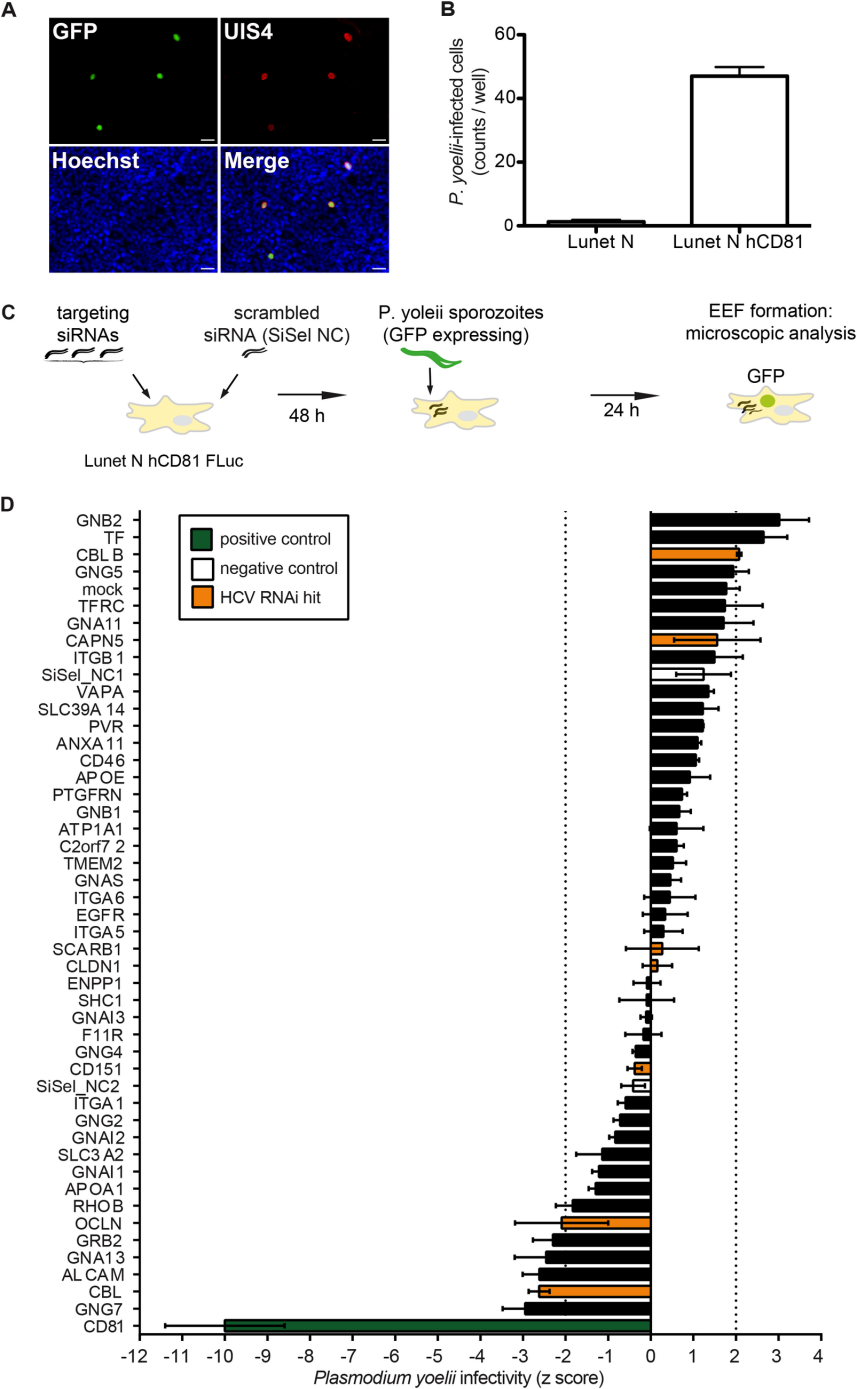
*Plasmodium* sporozoites use CD81, but not CAPN5 or CBLB for hepatoma cell entry. (A) Lunet N hCD81 human hepatoma cells were infected with sporozoites of a *P*. *yoelii* GFP reporter strain for 180 min, then fixed at 48 hpi, stained for the parasite protein UIS4 and the nuclear stain Hoechst 33342 and analyzed by fluorescence microscopy. Development of exoerythrocytic forms indicated by co-localization of GFP with the parasitophorous vacuole marker UIS4. Scale bar: 50 μm. (B) Lunet N and Lunet N hCD81 cells were infected as in (A) and productively infected cells quantified by fluorescence microscopy. Mean and SEM of 4 biological replicates shown. (C) Schematic overview of the experimental setup used to analyze the role of CD81 interacting proteins in *P*. *yoelii* entry into Lunet N hepatoma cells. (D) Human hepatoma cells were transfected with a pool of three siRNAs as described in Fig 3C, followed by infection with sporozoites of a *P*. *yoelii* GFP reporter strain. Infectivity was measured 24 hpi as formation of exoerythrocytic forms by microscopy. Knock down of CD81 significantly decreased *P*. *yoelii* infection (p≤ 0.05; abs (z score) ≥ 2). Data from 2 biological replicates shown as mean +SEM. See also S4 and S7 Figs.

Next we set out to understand whether a similar or distinct set of CD81 PPIs was necessary for hepatoma cell entry of the malaria parasite *Plasmodium yoelii*. Here, we used *P*. *yoelii* as surrogate system for the human pathogenic *P*. *falciparum*, since both species share the requirement for CD81 and P. *yoelii* can infect human hepatoma cell lines, while *P*. *falciparum* only infects primary cells [4,32]. First, we evaluated the *P*. *yoelii* requirement for CD81 using Lunet N hCD81 cells. As expected sporozoite entry and development of exoerythrocytic forms (EEFs) was strictly dependent on the expression of CD81 (Fig 4A and 4B). In contrast, *P*. *berghei* did not depend on CD81 and infected Lunet N cells efficiently, when SCARB1 was functional. Blocking of SCARB1 with a neutralizing antibody, however, led to CD81-dependent *P*. *berghei* sporozoite entry. This confirms a redundant role for CD81 and SCARB1 in *P*. *berghei* liver infection [33] (S4 Fig). Using CD81-dependent *P*. *yoelii* sporozoites and a similar RNAi setup as for HCV (Fig 4C), only CD81 was identified as important host factor required for sporozoite entry in this screen. Silencing of CD81 more pronouncedly affected *P*. *yoelii* infection as compared to HCV infection, which suggests a higher threshold level of CD81 required for *P*. *yoelii* infection [25,32]. Neither CAPN5 nor CBLB knockdown reduced *Plasmodium* sporozoite entry significantly, however CBL and GRB2, which are known HCV entry facilitators [15,29], reduced sporozoite entry twofold (Fig 4D). Thus, despite the usage of CD81 as entry factor by HCV and *Plasmodium* sporozoites, a distinct subset of CD81 interactors seems to aid entry into liver cells.

### CRISPR/Cas9 knockout identifies CAPN5 and CBLB as HCV entry facilitators

**Fig 5:**
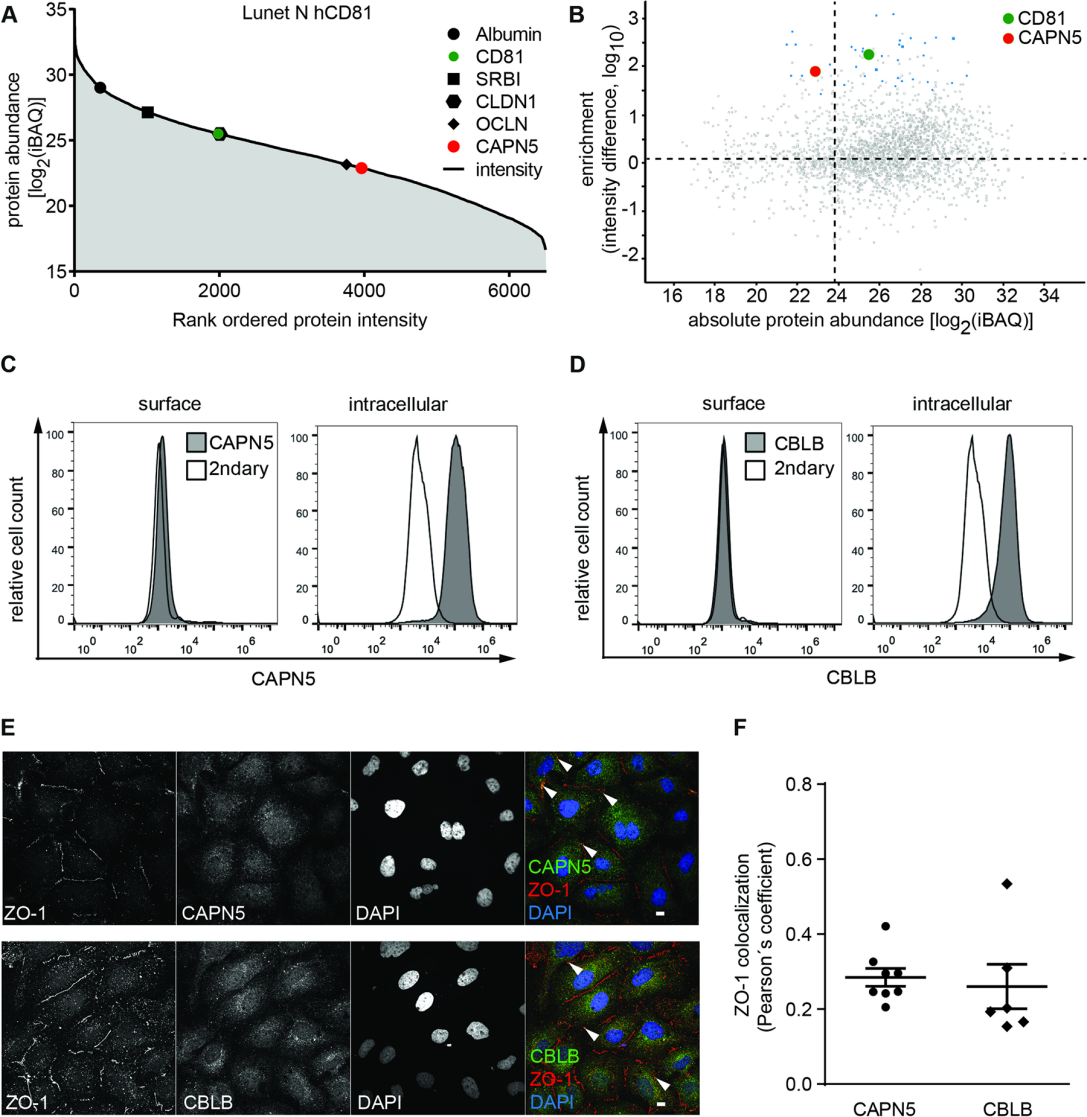
CAPN5 and CBLB are cytoplasmic proteins enriched in the CD81 complex. (A) Whole cell proteome quantification for Lunet N hCD81 cells. Expression level as iBAQ value indicated for the CD81 interactor CAPN5 (red) and the HCV entry factors CD81 (green), SCARB1 (black square), CLDN1 (black hexagon) and OCLN (black diamond). Albumin (black dot) shown as additional positive control. (B) Comparison of protein abundance in whole cell lysates and protein enrichment in CD81 co-IPs from Lunet N hCD81 cells. CAPN5 (red) and CD81 (green) are highlighted. Dotted lines indicate median values of all detected proteins. (C, D) Flow cytometric staining of CAPN5 and CBLB on the surface of naïve Lunet N hCD81 cells or after membrane permeabilization reveals intracellular localization of CAPN5 and CBLB (E) A subfraction of CAPN5 and CBLB colocalizes with the membrane marker ZO-1. Lunet N CRISPR scrambled cells were stained with anti-ZO-1 and anti-CAPN5 (upper panel) or anti-CBLB (lower panel). Nuclei were stained with DAPI. Arrowheads indicate colocalization of ZO-1 and CAPN5 or CBLB. Representative confocal images; scale bars 10 μm. (F) Pearson’s correlation coefficient for ZO-1 and CAPN5 or CBLB calculated by intensity correlation analysis. Each symbol represents an individual frame; horizontal lines indicate the mean ± SEM.

We prioritized CAPN5 and CBLB for follow up analysis for three reasons. Both proteins were neither reported as CD81 interactors nor as HCV entry facilitators previously. Moreover, CAPN5 and CBLB appear to be strongly associated with the CD81 complex as their total abundance in human hepatoma cell lysates was comparably low as quantified by intensity based absolute quantification (iBAQ). LC-MS/MS analysis of Lunet N hCD81 whole cell lysates revealed moderate expression levels for CAPN5 (log_2_(iBAQ) = 23) (Fig 5A). Expression was comparable to OCLN (log_2_ (iBAQ) = 23) and lower than for SCARB1, CLDN1 and CD81 (log_2_ (iBAQ)>25). Similar expression patterns occurred in all other tested human hepatoma cell lines (S5A and S5B Fig, S4 Table). CBLB, which we included as in silico predicted network edge, was undetectable by LC-MS/MS. However, we could clearly show protein expression in hepatoma cells by flow cytometry, immunofluorescence microscopy and immunoblot (Figs 5D, S5D and S5H). Comparison of the absolute CAPN5 abundance with the enrichment from the CD81 IP (Fig 5B) suggests that a large fraction of CAPN5 is associated with CD81 in human hepatoma cells.

Next, we addressed the subcellular localization of CAPN5 and CBLB. Both proteins have multiple cellular component annotations including plasma membrane, cytosol and nucleus. In human hepatoma cells we detected both proteins in intracellular compartments but not exposed to the cell surface (Fig 5C and 5D). Immunofluorescence analysis further confirmed expression in cytoplasmic and nuclear compartments as reported for other cell types (S5C and S5D Fig). Both CAPN5 and CBLB showed a weak co-localization with the plasma membrane expressed protein zonula occludens 1 (ZO-1) with Pearson’s correlation coefficients of 0.28 and 0.26, respectively (Fig 5E and 5F).

**Fig 6:**
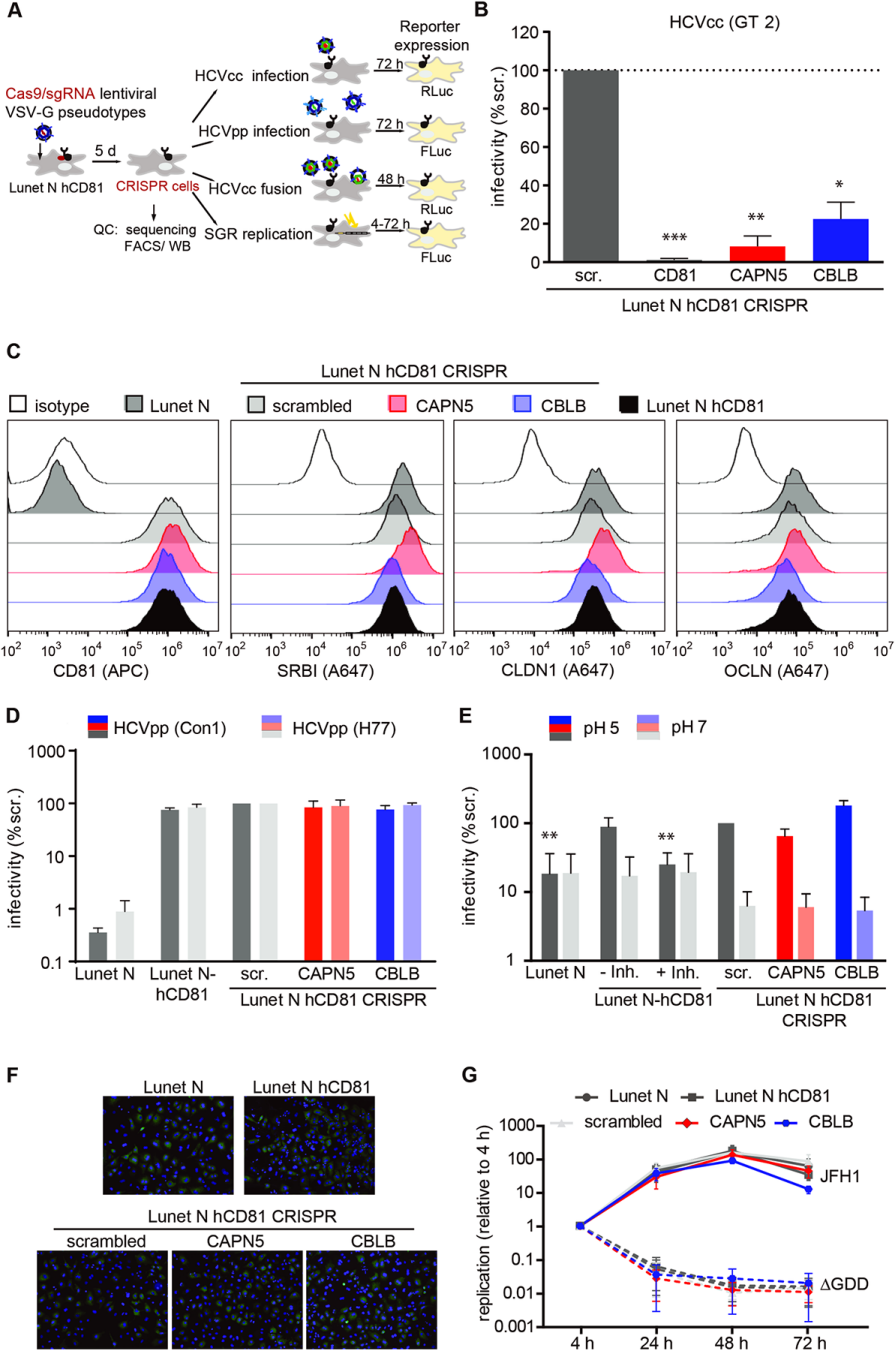
CAPN5 and CBLB support a postbinding step during HCV lipoviroparticle entry. (A) Schematic overview of the experimental setup used to analyze different steps of the HCV life cycle in the CRISPR/Cas9 knockout cell lines. (B) Infection of CAPN5 (red) and CBLB (blue) knockout and parental cell lines with HCV genotype 2 reporter virus. 72 hpi infection rates were quantified as luciferase activity and normalized to infection rates in cells transduced with a non-targeting scrambled sgRNA. CD81 knockout cells served as positive control. Data from 3 independent experiments shown as mean +SEM. (C) Flow cytometric surface staining of CD81, SCARB1, CLDN1 and OCLN in cells knocked out for CAPN5 (red) or CBLB (blue). Parental cells (black) served as positive control. Isotype control stainings or stainings with secondary antibody only (white) as negative controls. (D) Entry of lentiviral particles pseudotyped with glycoproteins from HCV GT1a (strain H77) or GT1b (strain Con1). Infectivity normalized to particles without envelope protein (negative control), to particles with VSV-G envelope (positive control) and to infection of cells transduced with non-targeting scrambled sgRNA. (E) Quantification of HCV fusion activity at the plasma membrane. Cells were pretreated with concanamycin A to inhibit endosomal acidification, cold-bound with HCV luciferase reporter virus (JcR-2A; 4°C, 2 h), shifted to 37°C (1 h) and washed with a pH 5 buffer to induce artificial plasma membrane fusion. A pH 7 buffer wash served to determine the background infection rate. 48 hpi infection rate was quantified as luciferase activity. Inh: flunarizine; scr: scrambled sgRNA (F) Immunofluorescence staining of cell lines electroporated with a HCV subgenomic replicon RNA (JFH1) at 48 hpt. Green: NS5A. Blue: DAPI. 10x magnification. (G) Cell lines were electroporated with wildtype HCV subgenomic replicon RNA (JFH1) or a polymerase active site mutant JFH1-ΔGDD (dotted lines), both encoding a luciferase reporter. Replication quantified as luciferase activity at the indicated time point post electroporation. Results normalized to the 4 h time point to account for electroporation efficiency. Data from at least three independent experiments shown as representative results (C, F) or as mean ± SEM (B, D, E, G). Significance according to unpaired t-test (B, E) or to MANOVA (G) indicated by * (p≤ 0.05), ** (p≤ 0.01), *** (p≤ 0.001). See also S6 Fig.

To further verify a role for CBLB and CAPN5 in HCV infection, we generated CRISPR/Cas9 knockout cell lines lacking CAPN5 or CBLB expression (Fig 6A). Control knockout cell lines with CD81 targeting and non-targeting single guide RNA (sgRNA) (scrambled) were generated in parallel. Genomes of CAPN5 and CBLB knockout cells were edited at the expected position (S5E and S5F Fig). Knockout cells displayed reduced expression levels of CAPN5, CBLB, or CD81 as demonstrated by immunoblotting (S5G and S5H Fig). We infected CD81, CAPN5, CBLB, and scrambled knockout cell lines with luciferase reporter cell culture virus (HCVcc, genotype 2a) and observed a ten-fold and five-fold reduction of HCV infection rates upon CAPN5 and CBLB knockout compared to the scrambled control, respectively. Knockout of CD81 almost completely abrogated susceptibility of human hepatoma cells to HCV (Fig 6B).Taken together we find that the CD81 interaction partners CAPN5 and CBLB are HCV host dependency factors.

### CAPN5 and CBLB support a postbinding step during HCV lipoviroparticle entry

Our findings that CAPN5 and CBLB form a complex with the HCV receptor CD81 (Figs 1E, 2E and 3A) together with the observation that both proteins are required for productive HCV infection (Fig 3C) raised the notion that they function during virus entry. To experimentally pinpoint which step in the HCV life cycle and specifically during HCV entry both proteins affected, we assessed HCV receptor expression, receptor usage, virus membrane fusion, uncoating and genome replication in CAPN5 and CBLB knockout cells (Fig 6A).

HCV entry critically depends on the four transmembrane proteins SCARB1, CD81, CLDN1 and OLCN. To exclude that CAPN5 or CBLB served as entry factor chaperones or regulators, we monitored surface expression levels of SCARB1, CD81, CLDN1 and OLCN in CAPN5 and CBLB knockout cells by flow cytometry. Neither CAPN5 nor CBLB affected surface expression of SCARB1, CD81, CLDN1 and OLCN on human hepatoma cells (Fig 6C). Similarly, EGFR surface expression levels remained unaffected by CAPN5 and CBLB knockout (S6A Fig). Next, we analyzed whether receptor usage of HCV was impaired by CAPN5 or CBLB. Therefore, we took advantage of lentiviral pseudoparticles displaying the HCV surface glycoproteins E1 and E2 and thus mimicking the receptor dependent entry steps [16,34]. Neither CAPN5 nor CBLB influenced cell entry of HCV glycoprotein pseudotyped lentiviruses (Fig 6D). This held true for both tested HCV genotypes, namely GT1a (H77 strain) and GT1b (Con1 strain). Expectedly, cells lacking CD81 expression showed drastically reduced HCV pseudotype entry.

The final steps in the HCV entry process are pH-dependent fusion of viral envelope and endosomal membrane and subsequent uncoating of the viral nucleocapsid. HCV membrane fusion is in part mimicked by lentiviral pseudotypes [16,17,35] and thus our data suggest that CAPN5 and CBLB are dispensable during this entry step. To further investigate a possible role of both proteins in fusion and uncoating, we fused HCVcc particles at the plasma membrane of CAPN5 and CBLB knockout cells by low pH wash and monitored infection rates. In this assay, entry through the natural route is prevented by concanamycin A blockage of endosomal acidification. CAPN5 and CBLB knockout cells displayed similar infection rates through the plasma membrane bypass, while the HCV fusion inhibitor flunarizine reduced infection rates to background levels (Fig 6E) [36]. This indicates that HCV membrane fusion is not altered by CAPN5 and CBLB and moreover that HCV uncoating is unaffected by CAPN5 and CBLB.

To address HCV postentry steps, i.e. genome translation and replication, in an entry independent assay, we transfected cells with HCV subgenomic RNA (Fig 6A). Transfection efficiencies were comparable in all tested cell lines (Fig 6F). Replication rates were not significantly affected by CAPN5 or CBLB knockout (Fig 6G). We observed a slightly reduced replication at 72 h post transfection in the CBLB knockout cells. A replication deficient subgenome of HCV (delta GDD) failed to replicate in all cell lines as expected. Subgenomic genotype 1b and full length genotype 2a replicons showed comparable results as genotype 2a subgenomes (S6B and S6D Fig). Lastly, we tested whether CAPN5 or CBLB affect assembly and release of new virions from the cell. Infectivity in supernatants of full length RNA genome transfected cells was largely independent of the presence of CAPN5 or CBLB (S6E and S6F Fig). A slight reduction in infectious titers was observed for CAPN5 knockout cells. Together, these data suggest that CAPN5 and CBLB affect primarily a post-binding but pre-fusion life cycle step, which is not mimicked by lentiviral pseudoparticles.

### CAPN5 and CBLB are HCV-specific, pan-genotypic entry facilitators with scaffolding function

**Fig 7:**
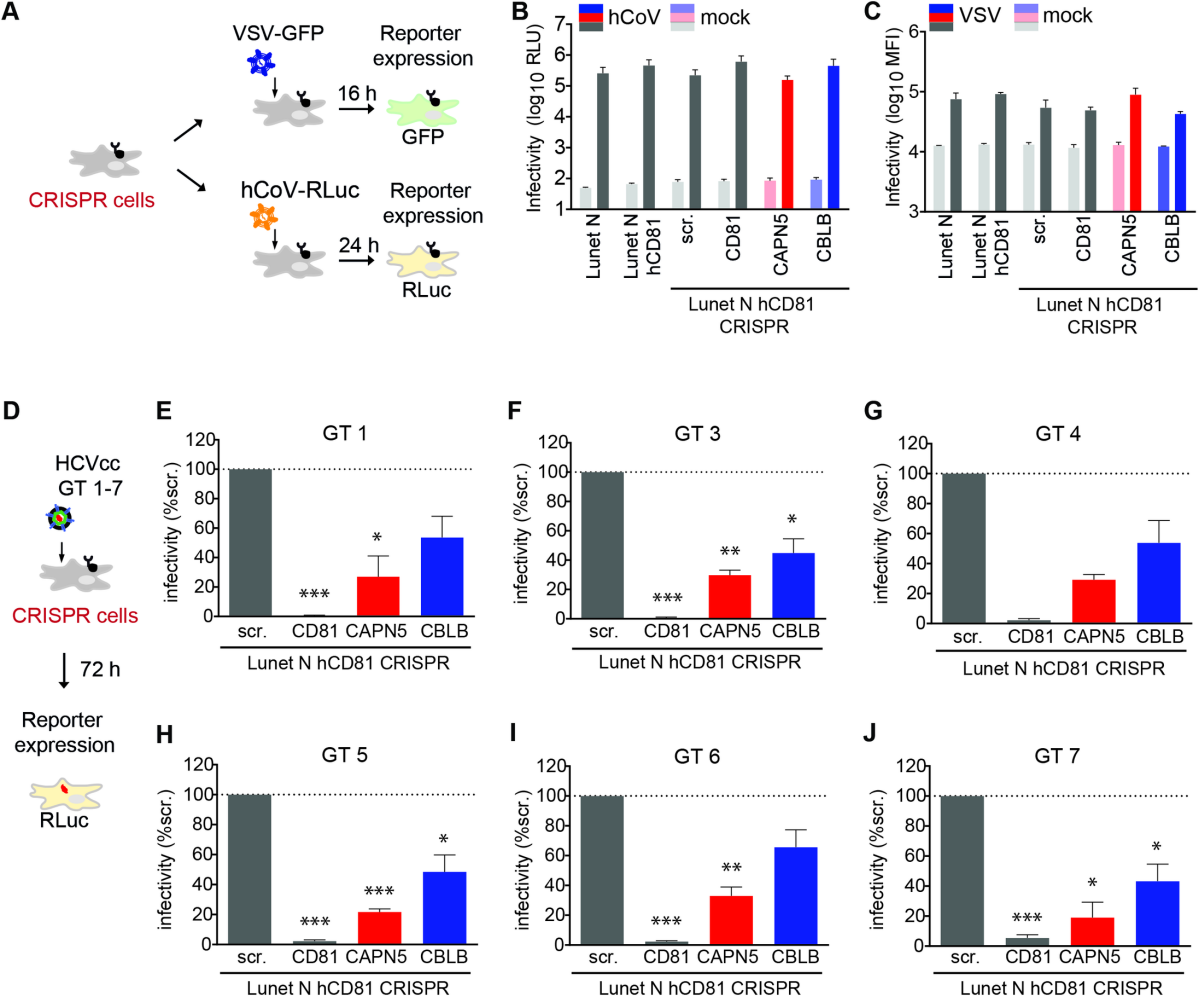
CAPN5 and CBLB are HCV-specific, pan-genotypic host factors. (A) Schematic overview of the experimental setup used to infect CRISPR/Cas9 knockout cell lines with human coronavirus (hCoV) and vesicular stomatitis virus (VSV). (B) Infection with hCoV expressing a luciferase reporter. Infectivity quantified 24 hpi as luciferase activity (RLU, relative light units). (C) Infection with VSV encoding a GFP reporter. Infectivity analyzed 16 hpi by flow cytometry as mean fluorescence intensity (MFI). (D) Schematic overview of the experimental setup used to infect CRISPR/Cas9 knockout cell lines with HCVcc intergenotypic chimeras expressing the structural proteins of genotypes 1 and 3–7. (E-J) Infection of CRISPR/Cas9 knockout and parental cell lines with chimeric HCV expressing glycoproteins from genotype (GT) 1 and 3–7. Infectivity measured 72 hpi as luciferase activity and normalized to infection of Lunet N hCD81 cells transduced with non-targeting scrambled sgRNA. Data from three independent experiments shown as mean +SEM. See also S6 Fig. Significance according to unpaired t-test (B, C, E-J) indicated by * (p≤ 0.05), ** (p≤ 0.01), *** (p≤ 0.001). Scr: scrambled sgRNA.

**Fig 8:**
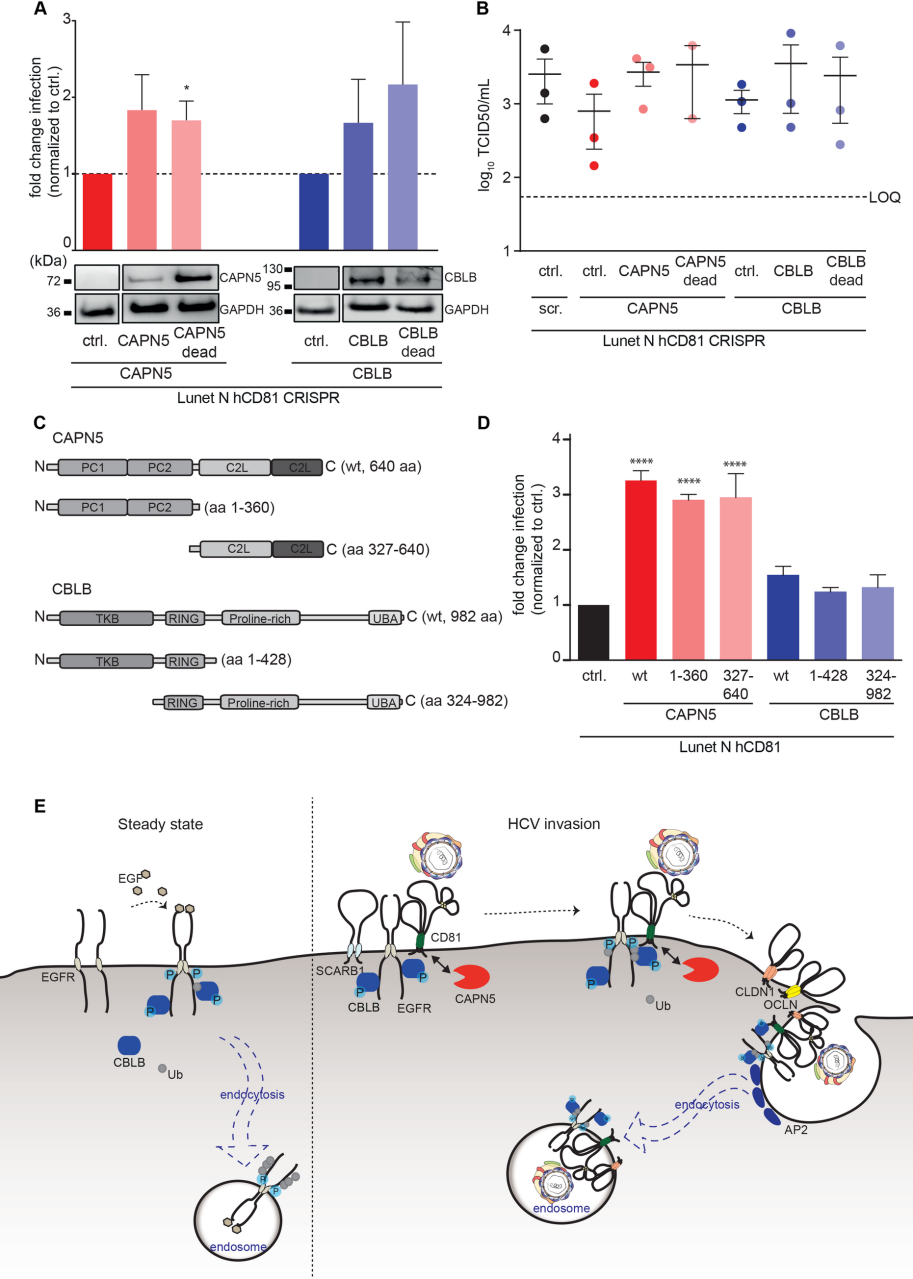
CAPN5 and CBLB have a scaffolding function in HCV infection. (A) CAPN5 (red) or CBLB (blue) knockout cells were complemented with sgRNA resistant CAPN5 (CAPN5; orange) / CBLB (CBLB; purple) or catalytically inactive CAPN5 (CAPN5 dead; light orange) / CBLB (CBLB dead; light purple), respectively. Infection of knockout and complemented cell lines with HCV genotype 2 reporter virus (upper panel). 72 hpi infection rates were quantified as luciferase activity and normalized to infection rates in knockout cells. Data from 3 independent experiments shown as mean +SEM. The protein expression level in knockout and complemented cell lines was analyzed by immunoblot (lower panel). Representative of 3 independent experiments. (B) HCV non-reporter infection in CAPN5 and CBLB knockout cells with and without complementation. Lunet N hCD81 cells with the indicated CRISPR and complementation construct were infected with non-reporter genotype 2 HCV (strain Jc1) and release of infectious particles measured at 72 h post infection by TCID50 assay. LOQ: limit of quantification. Shown are three (two for CAPN5 dead) independent experiments with technical duplicates each. (C) Schematic representation of CAPN5 and CBLB domain mutants. (D) The indicated truncated variants of CAPN5 and CBLB were overexpressed in Lunet N hCD81 cells and cells infected with *Renilla* reporter HCV. Infection was quantified 72 hpi by luciferase assay. PC: protease core; C2L: C2-like, TKB: tyrosine kinase binding; UBA: Ubiquitin associating; ctrl: empty vector control; scr: scrambled sgRNA. Bar graph shows mean + SD of one representative biological replicate (of four in total) with three technical replicates. Statistical analysis performed by ANOVA; ****p value<0.0001. (E) Model for the role of CAPN5 (red) and CBLB (blue) in HCV entry. Ub: ubiquitin, P: phosphate group. AP2: adaptor protein complex 2.

To assess whether CAPN5 and CBLB supported a virus life cycle step shared by RNA viruses replicating in cytoplasmic compartments, we infected knockout cells with human coronavirus (hCoV; strain 229E) and vesicular stomatitis virus (VSV; strain Indiana) (Fig 7A). CAPN5 and CBLB were dispensable for CoV and VSV infection in human hepatoma cells (Fig 7B and 7C). Similarly, *P*. *yoelii* and *P*. *berghei* sporozoites entered CAPN5 and CBLB knockout cells at least as efficiently as control cells (S7 Fig). In contrast, CAPN5 and CBLB were necessary for full infection with all tested HCV genotypes (Fig 7D–7J), revealing a pan-genotypic requirement for CAPN5 and CBLB. Complementation of CAPN5 and CBLB knockout cells with either enzymatically active or dead variants of both proteins led to a twofold increase in HCV susceptibility (Fig 8A). We confirmed these findings using non-reporter genotype 2 HCV (strain Jc1). CAPN5 and CBLB knockout cells showed a 3-fold drop in titers of released HCV, which was rescued by either wildtype or active site mutants of CAPN5 and CBLB (Fig 8B). To further shed light on CAPN5 and CBLB domains required for HCV infection, we overexpressed full length and truncated domain mutants of CAPN5 and CBLB in Lunet N hCD81 cells (Fig 8C). Overexpression of full length CAPN5, the N-terminal protease core domains (PC1 and PC2) or the two C-terminal C2-like domains (C2L) increased basal HCV infection three-fold. Overexpression of CBLB full length, N-terminal tyrosine kinase binding (TKB) and RING finger domain or the RING finger domain together with the C-terminal proline-rich and ubiquitin associating domain (UBA) led to a maximum 1.5-fold increase in HCV infection (Figs 8D and S8). This suggests that CAPN5 and CBLB function in HCV entry independent of their enzymatic and signaling functions, i.e. that both proteins have a scaffolding function. Taken together our study reveals a role for CD81 PPIs in productive HCV entry and specifically unravels CAPN5 and CBLB as host entry facilitators guiding productive uptake of HCV lipoviroparticles into replication competent intracellular sites.

## Discussion

Our study identifies CAPN5 and CBLB as components of the CD81 receptor complex and as HCV entry facilitators. We demonstrate that HCV binding to CD81 on the liver cell surface is not an isolated event, but steady state CD81 protein interactions are required for virion uptake. Specifically, we mapped protein interactions of CD81 in resting human hepatocytes and demonstrate that a subset of preexisting CD81 interactions is necessary for HCV infection.

### CD81 organizes tyrosine kinase and small G protein signaling networks in the membrane of human liver cells

Tetraspanins organize membrane microdomains and signaling platforms in a cell type specific manner. In B lymphocytes CD81 serves as a clamp for the B cell receptor complex [37,38]. In liver cells only little information on the CD81 guided membrane protein complexes is available and this is reflected by a limited knowledge on endogenous CD81 functions in the liver [15,39]. Here, we identified 33 CD81 associated proteins and their relative strength of interaction with CD81 as determined by abundance in CD81 co-IPs (depicted in a centered network in S3A Fig) plus nine closest neighbor network proteins. Using STRING network, Ingenuity pathway and DAVID GO enrichment analysis of the 42 CD81 network proteins in liver cells, we found ephrin receptor signaling, ephrin B signaling, thrombin signaling, and Tec kinase signaling as top four biological pathways (-log(p)>20) (S3B Fig). Notably, ephrin receptor serves as entry facilitator for HCV and other viruses such as Nipah virus and Kaposi sarcoma herpesvirus [14,40,41]. The top four associated diseases and disorders reflect a reported role of CD81 in cell migration and include organismal injury (33 molecules), cancer (28 molecules), inflammatory response (17 molecules) and infectious disease (15 molecules) (S3C Fig). The notion of an involvement of CD81 receptor complexes in cell migration is further strengthened by the association of the CD81 interactome with the following molecular and cellular functions: cell-to-cell signaling (33 molecules), cell movement (25 molecules) and cell morphology (23 molecules) (S3D Fig). The two top ranked cellular networks associated with the CD81 interactome in liver cells are cell-to-cell signaling and movement (score 36, 18 focus molecules) and infectious disease (score 16, 10 focus molecules) (S3E Fig). The analysis suggests a role for CD81 in integrin and G protein coordination as well as TFRC and EGFR signaling. The latter two receptors are HCV entry facilitators, highlighting once more that the here-defined CD81 interactome can indeed reveal host factors with a role in pathogen invasion in the liver.

### The CD81 network protein CBLB as virus entry facilitator

The here reported HCV host factor CBLB is an E3-ubiquitin ligase, which functions together with CBL [42]. CBLB and CBL regulate EGFR endocytosis by ubiquitination of the cytosolic receptor domains [43,44]. Notably, EGFR and CBL are known HCV entry facilitators [14,29]. Our analysis suggests that EGFR, CBL and CBLB form a complex with CD81 on liver cells and that CBL and CBLB are necessary for full HCV susceptibility. We found that the E3 ligase activity of CBLB is dispensable for promoting HCV uptake. This is in line with an E3 ligase activity independent role of CBLB in B cell receptor internalization [45]. CBLB is known to coordinate EGFR internalization together with CBL, which others and we describe as HCV entry facilitator [29,46]. This together with our findings suggests that CBLB has a scaffolding function during EGFR driven endocytosis of the HCV–CD81 platform. Future studies possibly using recently developed organoid models and single particle tracking of HCV may shed light on the spatiotemporal role of CBLB in HCV entry [13].

In this study, certain previously known HCV entry facilitators such as EGFR, SHC1, GRB2 and TRFC did not influence HCV susceptibility upon knockdown under the given assay conditions. However, we did not control silencing efficiency and used serum containing conditions, therefore especially growth factor and transferrin dependent entry facilitators presumably escaped detection. It is thus important to note that the RNAi screen may have missed CD81 interacting proteins with a role in HCV or *P*. *yoelii* infection.

CBLB and CBL were not identified as CD81 interacting partners under the given experimental conditions. Presumably, transient binding of E3 ligases and subsequent targeting of the protein complex to proteasomal degradation hampered the detection. Ubiquitin ligase substrate trapping methods may overcome such experimental limitations [47]. In this study, we could overcome this caveat by in silico network analysis and inclusion of closest network nodes in the functional validation.

The model of CD81 linked signaling platforms serving as entry microdomains is in line with recent reports on influenza virus entry. Influenza virus interacts with sialylated molecules on the cell surface, which causes clustering of lipid rafts and activation of raft-associated signaling molecules including EGFR. The latter then activates its endocytosis together with influenza virus [48]. It will be interesting to investigate whether CBL and CBLB affect influenza virus entry in a similar manner as HCV entry. Tetraspanins moreover play a role in entry and spread of HIV-1, human papillomaviruses and human cytomegalovirus [49–52]. Thus our study provides an important dataset of PPIs, which has implications beyond HCV infection. In fact we think that the unbiased interaction proteomics provides a different perspective on virus entry than functional knockout screens, as it opens avenues for diverse functional follow up studies and hypothesis generation.

In the absence of infection CD81 is thought to regulate migration of liver cells [39]. In confirmation of this notion, in silico analysis maps here identified CD81 network proteins to cellular movement pathways. Notably, expression of the CD81 interactor and HCV entry facilitator CBLB is altered in gastric and breast tumor tissue and CBLB regulates cell migration and epidermal to mesenchymal transition (EMT) through EGFR degradation [43]. This in line with recent reports that HCV entry facilitators like E cadherin can contribute to EMT [53]. Progression of HCV infected tissue to liver cancer is a slow, indirect and multifactorial process. Our network analysis suggests that cellular PPIs engaged by the virus during entry might be linked to tumor development. Specifically, HCV entry processes occurring in an infected liver may contribute to EMT and in the long run tip the balance towards tumor formation.

### CAPN5 forms a complex with CD81 and is required for productive HCV uptake

CAPN5 is a calcium dependent endopeptidase, which is predicted to regulate protein function through limited proteolysis [54]. Expression in lung, kidney and brain was previously reported and we demonstrate CAPN5 expression in primary hepatocytes and hepatoma cells [55]. Moreover, we show that CAPN5 associates with CD81 protein complexes and is required for HCV entry. Our data suggests that the putative endopeptidase function of CAPN5 is dispensable for HCV infection and that CAPN5 has a scaffolding function. Of note, CAPN5 is poorly characterized and its cellular functions as well as substrates remain enigmatic. Our work shows that CAPN5 has a function in HCV entry, which is independent of the predicted catalytic triad. Whether endogenous functions of CAPN5 are mediated by the predicted endopeptidase function, remains to be investigated.

Our findings that CAPN5 and CBLB are necessary for HCV infection but not for HCV translation, replication, assembly and release of virions strongly indicate a role in virus entry. Specifically, CAPN5 and CBLB regulate an entry step, which is not mimicked by lentiviral pseudoparticles. Two major differences between lentiviral pseudoparticles and authentic HCV particles exist. Firstly, HCV in contrast to lentiviral pseudoparticles tightly associates with serum lipoproteins to form lipoviroparticles [56,57]. Secondly, HCV replicates in specialized cytoplasmatic compartments while lentiviruses replicate in the nucleus [58]. Our data points towards a role of CAPN5 and CBLB in guiding endosomal uptake and trafficking to HCV replication competent cytoplasmic compartments. This model is supported by the notion that endosomal sorting depends on membrane protein–lipid complexes and that correct endosomal routing is critical for productive virus infection [59–61]. Thus we propose a model in which CBLB and CAPN5 regulate endosomal uptake of HCV–receptor complexes and subsequent routing towards replication competent subcellular compartments (Fig 8E).

### *Plasmodium* sporozoites use a distinct subset of CD81 interactors for liver cell entry

Our data confirms that HCV and the malaria parasite *Plasmodium* use distinct CD81-mediated entry pathways for liver cell invasion. This is in line with the fact that HCV critically relies on the host cell endocytic machinery, while *Plasmodium* is a motile organism that enters cells actively using its own motor machinery. Moreover, different regions in the CD81 ectodomain are required for HCV and *Plasmodium* entry, respectively [5,8,62–64]. In line with this, we demonstrate that CAPN5 and CBLB promote HCV entry, but not *Plasmodium* entry into human hepatoma cells. Interestingly, growth factor receptors are implicated in the entry process of HCV and *Plasmodium* [14,65], suggesting that some CD81 interaction partners may be hijacked by both pathogens. In this study, we only identified only CBL as common entry facilitator and in fact activation of growth factor receptors likely differs between both pathogens. While the *Plasmodium* thrombosponin-related anonymous protein (TRAP) can directly activate host kinases [66], HCV probably induces kinases through CD81-mediated receptor clustering. Such mechanistic details, however, remain to be elucidated. Interestingly, EGFR associated molecules scored positive for HCV and *Plasmodium* in the RNA interference screen. HCV relied on CBL and CBLB, while *Plasmodium* relied on CBL and the EGFR adaptor protein GRB2 [67]. GRB2 has also been reported as HCV entry facilitator [15]. We show that CBLB knockdown and knockout cells are fully functional for *Plasmodium* uptake. This highlights the versatile follow up of receptor interaction proteomics and demonstrates similarities and differences in usage of CD81 by HCV and *Plasmodium*.

### Implications for the understanding of liver pathogenesis and drug development

Viruses contribute to an estimated 30% of adaptive changes in the human proteome [68], suggesting that protective protein networks have evolved to fend off virus attacks on cells. Our RNAi data suggests that steady state interactions of the HCV receptor CD81 in liver cells are non-protective. In this study we concentrated on interactions occurring in susceptible primary liver cells and hepatoma cells. The latter were adapted to support the full HCV life cycle. Notably, we found a significant number of primary hepatocyte specific interactions, which we did not follow up on functionally in this study. It is conceivable that some of these interactions are protective. This, together with the stronger innate immune response of primary hepatocytes as compared to the highly HCV-adapted hepatoma cell lines, could explain the low susceptibility of primary cells to HCV. In infected patients a very slow disease progression with limited viral spread in the liver and symptoms occurring 15 to 25 years post contraction reflects this phenotype. Future work will clarify the existence of protective protein networks in primary hepatocytes. Clearly the CD81 interaction network in non-susceptible cells like B cells markedly differs from the here described liver cell CD81 network [69]. This confirms the notion that CD81 interactors like SCARB1 contribute to the narrow tissue tropism of HCV [70].

Systems biology approaches are proving increasingly valuable to understand how viruses cause disease [71]. Proteomics methods, in contrast to genomics and transcriptomics methods, have just in the past decade reached resolution and throughput necessary for systematic analysis of host pathogen interactions and overall proteome profiles of infected cells [72]. Importantly, protein interaction profiling in combination with computational biology is an iterative process which ultimately can map a near to completion cellular network. In the future, we may be able to predict in silico, how perturbations of such a network by pathogens influences cellular functions. In the current study, we describe the isolated network of the plasma membrane protein CD81. This tetraspanin functions in cell motility and in confirmation we found integrins, small G proteins, GTPases and growth factor receptors involved in cell migration as CD81 network molecules [39]. To our current knowledge, HCV uses only two of these molecules (EGFR, ITGB1) for cell entry and *Plasmodium* presumably none of the molecules involved in cell migration. Thus, design of antimicrobial drugs with minimal side effects on endogenous CD81 functions may be possible. Importantly, pathogens may use protein domains and functions, which are not required for the endogenous role of the protein, as indicated by the functionality of active site mutants of CAPN5 and CBLB in HCV entry. It may thus be feasible to specifically target protein functions used by pathogens and thereby exclude or minimize side effects. Furthermore, systems virology approaches using primary cell material from numerous donors hold the promise of elucidating individual differences in protein expression and networks, thereby spurring personalized medicine. In conclusion our study not only impacts the understanding of HCV entry and pathogenesis, but also sets the stage for further elucidation of HCV driven carcinogenesis and entry of the malaria parasite into the liver.

## Materials and methods

### Cells

Huh-7 Lunet N cells (subclone #3) used in this study were designated as Lunet N and are described in detail in [26]. All Lunet N derived cell lines were cultured at 37°C in Dulbecco’s modified Eagle medium (Invitrogen) supplemented with 10% FCS (FCS Gold; PAA, Coelbe, Germany), 2 mM L-glutamine (Invitrogen), 0.1 mM non-essential amino acids (Invitrogen), 10 U/ml Penicillin (Invitrogen) and 10 μg/ml Streptomycin (Invitrogen). Lunet N hCD81 and Lunet N hCD81 FLuc were cultured in the presence of 5 μg/ml Blasticidine (InvivoGen).

Primary human hepatocytes were isolated from liver specimens and plated in collagen-coated 6-well dishes at 1.3x106 cells/well as described in [73].

### Antibodies

The antibodies against β-actin (clone AC-15, Sigma), CAPN5 (Abcam), CBLB (Invitrogen and clone C-20, Sant Cruz), CD81 (clone JS-81, DB Pharmigen), CLDN1 (R&D), EGFR (clone LA1, Merck), GAPDH (Sigma), HA (clone 16B12, Covance), NS5A (clone 9E10, kindly provided by Charles M. Rice), OCLN (clone OC-3F10, Invitrogen), SCARBI (Novus) and ZO-1 (clone 1/ZO-1, BD Biosciences) were used for immunoblot, flow cytometry and immunofluorescence staining. Horseradish peroxidase (HRP)-coupled anti-mouse and anti-rabbit antibodies were from Sigma and Jackson Lab Inc., respectively. Alexa 488-conjugated secondary antibodies were from lifeTechnologies, Alexa 647-conjugated secondary antibodies were from Invitrogen, APC- or FITC-conjugated secondary antibodies were from eBioscience. For immunoprecipitation, we used antibodies against CD81 (clone 1.3.3.22, Santa Cruz), HA (clone 16B12, Covance) and a mouse IgG_1κ_ (clone MOPC-21, BD Pharmigen).

### Immunoblot, flow cytometry and immunofluorescent microcopy

For western blot analysis, equivalent volumes of cell lysates, IP flowthroughs or IP eluates were boiled 5 min with SDS sample buffer under non-reducing conditions, resolved by SDS-PAGE and transferred to PVDF membranes by electroblotting. Membranes were probed with primary antibodies o/n at 4°C, then with secondary HRP conjugated antibodies for 1 h at room temperature and analyzed using a chemiluminescence (Intas) system.

For flow cytometry, cells were trypsinized, quenched and surface stained with primary antibodies in PBS containing 1% FCS for 30 min on ice. After a brief wash, secondary Alexa647- or Alexa488-conjugated secondary antibodies were added for 30 min on ice. Cells were washed three times and analyzed on a FACSAccuri (BD Bioscience). Data analysis was performed using FlowJo.

For immunofluorescence analysis cells were plated on poly-lysine coated cover slips and fixed with 3% paraformaldehyde for 20 min. After three PBS washes, cells were permeabilized with 0.05% TX-100 for 4 min. Anti-CAPN5 and anti-CBLB antibodies were per-adsorbed over night at 4°C on CAPN5 and CBLB knockout cells, respectively. Scrambled knockout cells were then incubated o/n at 4°C with these pre-adsorbed antibodies followed by staining for 1 h at RT with secondary Alexa-fluorophore conjugated antibodies. DAPI counterstained cells were mounted on glass slides with ProLong Gold antifade mountant (Molecular Probes, W32466) and analyzed by confocal microscopy using an inversed confocal laser-scanning microscope (Olympus Fluoview 1000), using a 60x or 100x magnification lens. The three channels (blue, green, and red) were read in a sequential acquisition mode, with an average of 3 frames for each picture (Kalman *n* = 3). Data analysis was performed using FluoView (Olympus) and Image J. Intensity correlation analysis was performed using the Image J colocalization analysis plugin. Pearson’s colocalization coefficients were calculated for at least six individual frames.

### CRISPR/Cas9 knockout sgRNAs and primers

CRISPR/Cas9 knockout cells were generated as described in the main text. Table 2 lists the sgRNAs used to target the indicated genes. A scrambled, non-targeting sgRNA was used as control. To control disruption of the target genes, sgRNA binding regions were amplified and sequenced using the primers listed in Table 2.

**Table 2:** Sequence of sgRNAs and primers used for CRISPR/Cas9 knockout cell generation and evaluation.

Target gene	sgRNA sequence	Primer left	Primer right
CAPN5	CGTCAGTGGCGGGGAAGAGG	AAGCCCTATGAGGACCAGAACT	GTTTCAGCACCCTCACTTTCTC
CBLB	GTTGCACTCGATTGGGACAG	TTCTTTTGCTTGGAAGAAACCT	TCAATCAGGGCTTGAAATAAGG
CD81	TGGTGGTCTGCGGGTCATGG	-	-
scrambled	CTAAGGTTAAGTCGCCCTCG	-	-

### CRIPSR/Cas9 knockout cell generation and validation

Single guide RNA (sgRNA) sequences were selected using CHOPCHOP [98] and cloned into pLenti CRISPR v2 ccdB as described in [99,100]. Lentiviral VSV-G pseudotyped particles were generated using standard procedures in 293T cells and hepatoma target cells were transduced with lentiviruses for 4 h. 48 hpt cells were selected for Cas9 and sgRNA expression by puromycin selection. Surviving cells were characterized by flow cytometric antibody staining. Batch knockout populations were further validated by immunoblot and sequencing of the edited genomic DNA region.

### Cloning of CRISPR/Cas9 knockout and complementation constructs

For complementation of knockout cells, sgRNA-resistant CAPN5- and CBLB-constructs were generated. To this end, CAPN5 and CBLB were amplified and MluI and SpeI binding sites were added using the following primer pairs: 5’-AAAAAAAC GCGTGCCACCATGTTCTCGTGTGTGAAGCC-3’ and 5’-AAAAAAACTAGTTCAGACA GCCATGAGGGAGG-3’ for CAPN5 or 5’-AAAAAAACGCGTGCCACCATGG CAAACTCAATGAATGG-3’ and 5’-AAAAAAACTAGTCTATAGATTTAGAC GTGGGGATAC-3’ for CBLB. The sgRNA binding site resistance was generated using 5’-CATCTGTTGCTGGAAATAGGGGGTCCTCGAAGAGCACCT-3’ and 5’GGTGCT CTTCGAGGACCCCCTATTTCCAGCAACAGATG-3’ for CAPN5 or 5’-TTAAGCTG TACGCGGTTAGGCCAAGTGGCCATTGGCTATGTG-3’ and 5’-CCCATTGGCCTA ACCGCGTACAGCTTAACCGGAAAATATAGCTTCC-3’ for CBLB.

The sgRNA-resistant constructs were used to generate active site mutants. For CAPN5 the following primers were used to introduce mutations in the three active sites: 5’-GGTGGGCAACGCCTGGTTTGTGGCAGCCTGC-3’ and 5’-CCACAAACCAGGCGT TGCCCACCTGGCC (C81A), 5’-CGGCCTGGTAAAGGGCGCCGCATACGCCGTC-3’ and 5’-GCGTATGCGGCGCCCTTTACCAGGCCG-3’ (H252A), 5’-ATGATCC GCCTGCGCGCCCCCTGGGGCGAGCGG-3’ and 5’-CGCCCCAGGGGGCGCGCAGGC GGATCATGT-3’ (N284A). For CBLB the following primers were used to introduce the active site mutation: 5’-CTTTTCAGCTCGCAAAGATTTGTGCAGAGAATGACAAA-3’ and 5’-TTCTCTGCACAAATCTTTGCGAGCTGAAAAGTGGAGCCC-3’ (C373A).

### CAPN5 and CBLB truncation construct generation

CAPN5 constructs (CAPN5 amino acids 1–360 and 327–640) as well as CBLB constructs (CBLB amino acids 1–428 and 324–982) were designed to contain a tandem hemagglutinin (HA) tag at the C-terminus via a Gly_4_SerGly linker and ordered as gBlocks Gene Fragments (IDT, USA). DNA fragments were cloned into the pWPI_BLR vector using Gibson assembly according to manufacturer’s instructions (New England Biolabs, Ipswich, MA, USA).

### Viruses and pseudoparticles

HCV full length (JcR-2a, Jc1 and intergenotypic chimeras) [74–79] and subgenomic (JFH1, JFH1-ΔGDD) RNA was in vitro transcribed and used to transfect cells as previously described [80–82]. To analyze replication independent of entry, cells transfected with subgenomic replicons were lysed at 4–72 h post transfection and luciferase activity was measured. For infection, full length HCV stocks were generated as described in [19] and used to infect cells for 72 h followed by luciferase measurement in cell lysates. Jc1 infection was performed at MOI 0.1 and quantified by harvesting cell culture supernatants at 72 h post infection and subsequent titration on naïve Huh-7.5 cells by limiting dilution with an immunohistochemistry readout at 72 h post infection.

Recombinant VSV expressing GFP was kindly provided by Gert Zimmer [83]. Cells were infected with VSV-GFP for 16 h followed by quantification of the GFP signal via flow cytometry using the Accuri C6 (BD). Human Coronavirus expressing a *Renilla reniformis* luciferase was kindly provided by Volker Thiel and used to infect cells for 24 h followed by luciferase measurement in cell lysates [84].

Lentiviral pseudoparticles encoding a Firefly luciferase were generated as described in [19]. Subsequently, target cells were transduced with the pseudoparticles for 72 h, the cells were lysed and the luciferase signal quantified. Lentiviral pseudoparticles encoding sgRNA, Cas9 or coding sequences of CAPN5 or CBLB and carrying VSV glycoprotein [85] were generated using the same protocol. Subsequently, target cells were transduced with pseudoparticles and cells were cultured for further analysis.

### HCV fusion at the plasma membrane assay

To analyze fusion of HCV particles at the plasma membrane, we performed a fusion assay as described in [36]. In brief, cells were seeded at 2*10^5^ cells/well in poly-L-Lysine coated 6 well plates. Cells were pre-treated for 1 h with 5 nM concanamycin A to inhibit acidification of endosomes. Subsequently, JCR-2a (produced as described above) was allowed to bind for 2 h at 4°C in the presence of concanamycin A, virus was removed and cells were shifted to 37°C for 1 h in the presence of concanamycin A to induce priming of glycoproteins of bound virus. Afterwards, fusion was triggered by a 5 min wash with a pH 5 citric buffer; a pH 7 citric buffer was used as control. Cells were incubated for another 3 h with medium containing concanamycin A, then the medium was replaced and luciferase activity was measured 72 hpi. As control, the fusion inhibitor flunarizine was added at 4 μM in addition to concanamycin A.

### Production of soluble CD81-LEL (sEL2)

A soluble form of the CD81-LEL fused to glutathione-S-transferase (GST) was produced as described in [26]. In short, *Escherichia coli rosetta gami* were transfected with plasmids encoding the CD81-LEL-GST fusion protein and grown at 37°C to an OD_592_ = 0.8, followed by induction of protein expression by addition of 1 mM IPTG (Sigma Aldrich) for 4 h at 37°C. Subsequently bacteria were pelleted and lysed by freezing in liquid N_2_ and sonication. The GST-fusion proteins were recovered by affinity chromatography using 100 μl glutathione agarose (Sigma) at 4°C overnight. The next day, the agarose was washed three times with 0.5% Tween20 in PBS; GST-fusion proteins were eluted in three steps with 100 mM L-glutathione (Sigma) and dialyzed in PBS overnight at 4°C. The concentration of the eluted protein was determined by Bradford assay.

### Co-immunoprecipitation

One-step immunoprecipitations of membrane proteins were performed using aminolink plus protein A/G resin (Pierce) with covalently bound anti-CD81 (clone 1.3.3.22, Santa Cruz), anti-HA.11 (clone 16B12, Covance) or IgG_1_κ isotype control (clone MOPC-21, BD Pharmigen) antibodies. CD81 and associated proteins were eluted from the resin using glycine buffer (pH 2.8) and precipitated with ethanol, sodium acetate (pH 5) and glycogen as described elsewhere [86]. Experiments were conducted in four biological replicates from four independent hepatoma cell passages. Primary human hepatocyte co-IPs were performed as single experiments from seven independent donors. Efficiency of bait enrichment was determined for each hepatoma cell IP sample and for IPs from two primary cell donors by immunoblotting. All hepatoma cell IP eluates and IPs from primary cells of five independent donors were used for liquid chromatography tandem mass spectrometry (LC-MS/MS) analysis.

### Whole cell proteomes

Hepatoma cells were pelleted and lysed in 4% SDS, 10 mM HEPES (pH 8), 10 mM DTT. Cells were heated at 95°C for 10 min and sonicated at 4°C for 15 min (level 5, Bioruptor, Diagenode). Proteins were precipitated with acetone at −20°C overnight and resuspended the next day in 8 M urea, 10 mM Hepes (pH 8). Proteolytic digestion was carried out as described below and samples subjected to LC-MS/MS analysis.

### LC-MS/MS analysis

Protein samples were reduced, alkylated and trypsinized as previously described [19]. Tryptic peptides were separated on an EASY-nLC 1000 HPLC system coupled online to an orbitrap mass spectrometer (Q Exactive HF, Thermo Fisher Scientific) via nanoelectrospray source in single run experiments. Data was acquired using the Xcalibur software (Thermo Scientific).

MS raw files were analyzed using MaxQuant algorithms against the human Uniprot FASTA database and a common contaminants database (247 entries) by the Andromeda search engine^.^ with a false discovery rate (FDR) of 1% at peptide and protein level [87,88]. Inclusion criteria were set to at least one unique or razor peptide with a minimum length of 7 amino acids per protein group. MaxLFQ algorithms were used for protein quantification [89].

### Bioinformatics, hierarchical clustering, network analysis and hit scoring

Statistical analysis of proteomics data was conducted using a nonparametric two samples test correcting for multiple hypothesis testing in Perseus [90]. Proteins significantly enriched in CD81 co-IPs from hepatoma cells (FDR≤0.05, s0 = 1; s0: the minimal log_10_ fold change) and primary hepatocytes (intensity difference≥4) were considered for further analysis. 33 interaction partners of CD81 fulfilled this criterion. For functional follow up of CD81 binding proteins, we additionally included the nine closest nodes from a STRING network analysis based on experimental data and database search with a confidence of 0.4 [91] of the 33 proteins, resulting in 42 CD81 binding partners to be tested for their role in HCV infection. Hierarchical clustering and integrated network analysis including GO annotation were described previously [19]. Centered networks of the 42 hits were visualized using Cytoscape (version 3.5.0) and median intensity differences as measure of interaction strength [92,93].

The protein interactions from this publication have been submitted to the IMEx (http://www.imexconsortium.org) consortium through IntAct [94] and assigned the identifier IM-25678.

### RNAi screen for HCV host factors

Lunet N hCD81 cells stably expressing *Firefly* luciferase (Lunet N hCD81 FLuc) were transfected with pools of three siRNAs targeting the 42 selected CD81 interaction partners (Ambion Silencer Select) and infected with the *Renilla* luciferase reporter virus JcR-2A as detailed in [19]. The screen was conducted nine times on cells of three independent passages. Normalization and statistical analysis was performed on a set of 45 targets including CD81, CLDN1 and SCARB1 positive controls in R using the Bioconductor package RNAither [95]. Briefly, normalization for plate effects was done by subtracting the plate mean and dividing by the plate standard deviation.

### Plasmodium infection

GFP-expressing *P*. *yoelii* and *P*. *berghei* [96] were collected from the salivary glands of infected *Anopheles stephensi* mosquitoes. Lunet N or Lunet N hCD81 cells (1 x 10^4^ cells/well plated in 96 well plates 48 hours prior to infection) were co-incubated for 3 hours with sporozoites (5 x 10^3^ /well), then washed to remove extracellular parasites and further cultured for 24–36 hours. Infected cultures were analyzed by fluorescence microscopy, after fixation with 4% PFA and staining with antibodies specific for the parasitophorous membrane marker UIS4 [97] and the nuclear stain Hoechst 33342.

### Statistical analyses

Experiments were performed at least in three biological replicates with three technical replicates per experiment unless otherwise stated. Results are presented as mean plus the standard deviation (SD) of three biological replicates unless otherwise indicated. The 50% tissue culture infectious dose (TCID50) was calculated based on Kaerber and Spearman [101,102]. Statistical significance was determined by one-way Analysis of Variance (ANOVA) followed by Dunnett’s multiple comparison test or by unpaired t test in GraphPad Prism 5 (GraphPad Software, Inc., San Diego). A *p* value of less than 0.05 was considered statistically significant.

### Ethics

The ethics commission of Hannover Medical School approved the study on primary hepatocytes (vote # 3319–2016). All patients were adults and gave written informed consent. The study was performed in compliance with the ethical standards laid down in the 1975 Declaration of Helsinki.

## Supporting information

S1 Fig Characterization of Lunet N cell lines expressing different CD81-variants. (A) Schematic representation of CD81-variants used in this study to analyze the CD81-interactome in human hepatoma cells. (B) Immunoblot analysis of CD81-expression in Lunet N derived cell lines used in this study. Protein expression was analyzed with an anti-CD81 antibody. Parental Huh-7.5 cells served as positive control, Lunet N cells served as negative control. GAPDH served as loading control. (C) Flow cytometric staining of surface-expressed CD81 in Lunet N derived cell lines used in this study. Parental Huh-7.5 cells served as positive control, Lunet N cells served as negative control. (D) Immunofluorescence staining of CD81 in Lunet N derived cell lines used in this study and after fixation and permeabilization. Parental Huh-7.5 cells served as positive control, Lunet N cells served as negative control. Stainings were analyzed by confocal microscopy. Scale bar: 10 μm. (E) Entry of lentiviral particles pseudotyped with glycoproteins from HCV GT1a (strain H77). Lentiviral particles pseudotyped with the VSV envelope proteins or with no envelope proteins served as positive and negative control, respectively. Infectivity was analyzed 72 h post infection by luciferase measurement. (F) Infection with the HCV reporter virus JCR-2a or a Coronavirus (CoV). Infectivity was analyzed 72 h or 24 h post infection, respectively, by luciferase measurement. Data from three independent experiments shown as mean +/- SEM.
(TIF)

S2 Fig Protein enrichment in CD81-IPs from different hepatoma cell lines. (A) Dot plot showing LFQ intensities of proteins in CD81- and HA-IPs from the indicated cell line lysed with NP-40 containing buffer. CD81 (green) and SCARB1 (black) served as positive and APOL2 (white) as negative control. Median of 4 biological replicates. (B) Pretest for choice of detergents: Intensity differences of CD81 in CD81-IPs from Lunet N hCD81 compared to Lunet N. Cells were lysed with buffers containing Brij-58, Brij-97 or NP-40 prior to IP. Mean of four biological replicates. (C) Pretest for choice of detergents: Number of proteins found to be ≥ 4-fold enriched in CD81-IPs from Lunet N hCD81 compared to CD81-IPs from Lunet N lysed with the indicated detergents. Among these proteins, the number of membrane associated proteins, known CD81-interactors and HCV entry co-factors are plotted. Mean of four biological replicates. n.d. = not detected. (D) Dot plot showing LFQ intensities of proteins in CD81- and HA-IPs from the indicated experimental conditions. CD81 (green) and SCARB1 (black) served as positive and APOL2 (white) as negative control. Shown are median logarithmic protein intensities of 4 biological replicates in co-IPs from cells after incubation with indicated cross-linkers or CD81-LEL prior to lysis with Brij-58 containing buffer. (E) Volcano plot visualizing two-sample t-test comparing LFQ intensities of proteins found in CD81-IPs from Lunet N hCD81HA and Lunet N. For each protein the t-test difference (log_10_) of CD81 versus control co-IP of 4 biological replicates is plotted against the p value (-log_10_). FDR = 0.01; s0 = 2. Proteins significantly enriched are highlighted in dark grey. CD81 (green), SCARB1 (black) APOL2 (white) and CAPN5 (red) are highlighted. (F) Volcano plot visualizing two-sample t-test comparing LFQ intensities of proteins found in HA-IPs from Lunet N hCD81HA and Lunet N cells incubated with soluble CD81-LEL. For each protein the t-test difference (log_10_) of HA versus control co-IP of 4 biological replicates is plotted against the p value (-log_10_). FDR = 0.05; s0 = 1. Proteins significantly enriched are highlighted in dark grey. CD81 (green), SCARB1 (black) APOL2 (white) and CAPN5 (red) are highlighted. (G) Venn diagram showing the overlap of CD81 interacting proteins found in co-IPs from cells expressing hCD81 (green), hCD81HA (light red) or cells expressing hCD81HA and incubated with an excess of soluble CD81-LEL (purple).
(TIF)

S3 Fig CD81 Organizes tyrosine kinase and small G protein signaling networks in the membrane of human liver cells. (A) Centered network depicting the 42 CD81 associated proteins identified in this study. Each node represents one protein and the length of the edges reflects the median enrichment score for each protein in CD81 co-IPs from hepatoma cells and primary hepatocytes. The nine in silico predicted interaction partners were assigned an artificial score and are depicted in the periphery of the network. CD81 (green), CAPN5 (red) and CBLB (blue) are highlighted. (B) Ingenuity pathway analysis of 42 CD81 interaction partners. P value (left y-axis) and overlap with in total reported pathway molecules (right y-axis) shown. (C, D) Ingenuity pathway enrichment analysis for diseases and disorders as well as for molecular and cellular function. The number in the donut chart represent the number of proteins. (E) Top two Ingenuity pathway molecular networks for the 42 liver cell CD81 interaction partners. Proteins identified in this study highlighted in grey. Additional network nodes shown in white. All proteins placed in their approximate subcellular localization.
(TIF)

S4 Fig *P*. *berghei* can utilize hCD81 or hSCARB1 for hepatoma cell entry. Lunet N and Lunet N hCD81 human hepatoma cells were infected with sporozoites of a *P*. *berghei* GFP reporter strain for 180 min in the presence or absence of an anti-SCARB1 antibody, then washed and further cultured until fixation at 36 hpi. Exoerythrocytic forms were quantified by fluorescence microscopy. Mean and SEM of 4 biological replicates shown.
(TIF)

S5 Fig Expression and KO of CAPN5 and CBLB in hepatoma cell lines. (A, B) Whole cell proteome quantification for (A) Lunet N and (B) Lunet N hCD81-HA cells. Expression level as iBAQ intensity highlighted for the CD81 interactor CAPN5 and the HCV entry factors CD81, SCARB1, CLDN1 and OCLN. (C, D) Immunofluorescence staining of (C) CAPN5 and (D) CBLB in Lunet N hCD81 after fixation and permeabilization. Stainings were analyzed by confocal microscopy. Scale bar: 10 μm. (E, F) Sequencing of genomic DNA isolated from parental and KO cell lines. The (E) CAPN5 and (F) CBLB genes were amplified and sequenced in the indicated KO and control cell lines. The sgRNA binding sites are highlighted in blue. A frameshift in the CAPN5- and CBLB-genes is visible in the respective KO cells, while the genes are intact in all other cell lines. (G, H) Immunoblot analysis of (G) CAPN5- and (H) CBLB-expression in parental and KO cell lines. HepG2 cells served as positive control for CBLB-expression, GAPDH served as loading control.
(TIF)

S6 Fig CAPN5 and CBLB do not influence EGFR expression, HCV replication, HCV assembly and HCV release. (A) Flow cytometric surface staining of EGFR in cells knocked out for CAPN5 (red) or CBLB (blue). Parental cells (black) served as positive control. Isotype control stainings or stainings with secondary antibody only (white) as negative controls. (B) Schematic overview of the HCV subgenomic replicon encoding a firefly luciferase and the NS proteins from HCV GT 1b isolate Con1, which was used to electroporate the parental and KO cell lines. Replication was monitored at the indicated time point post electroporation by luciferase measurement. Results are normalized to the 4 h time point to account for electroporation efficiency. Data from at least three independent experiments shown as mean +/- SEM. (C) Schematic overview of the experimental setup used to transfect parental and KO cell lines with a HCV reporter virus (JCR-2a) and analyze replication, assembly and release of new virus particles, as shown in D-F. (D) Replication was analyzed in the cell lysates by measuring luciferase activity at 4–72 h post electroporation. (E) Assembly and release was analyzed by transferring supernatant of electroporated cells to naïve Lunet N hCD81 cells. Infectivity was determined by measuring luciferase activity in the target cell lysates at 4–72 h post infection. (F) Infectious particle release was normalized to viral genome replication. Data from one representative experiment (D-F).
(TIF)

S7 Fig CAPN5 and CBLB are dispensable for *Plasmodium* entry into hepatoma cells. Lunet N hCD81 human hepatoma cells carrying the indicated sgRNA were infected with sporozoites of *P*. *berghei* or *P*.*yoelii* GFP reporter strains for 180 min, then washed and further cultured until fixation at 36 hpi. Exoerythrocytic forms were quantified by fluorescence microscopy. Mean and SEM of 5 technical replicates shown. Experiment performed twice for *P*. *yoelii* and once for *P*. *berghei*.
(TIF)

S8 Fig Expression of truncated CAPN5 and CBLB variants in Lunet N hCD81 cells. Cell lysates of Lunet N hCD81 human hepatoma cells expressing the indicated domain mutant of CAPN5 and CBLB fused to a tandem HA tag were analyzed by immunoblot against the C-terminal HA-tag. GAPDH served as loading control.
(TIF)

S9 Fig Graphical summary. A combined label free quantification (LFQ)–RNA interference (RNAi)–CRISPR/Cas9 workflow identifies calpain-5 (CAPN5) and Casitas B-lineage lymphoma proto-oncogene B (CBLB) as hepatitis C virus (HCV) entry factors. CAPN5 and CBLB are critical host factors for all HCV genotypes and guide virus endocytosis and endosomal trafficking.
(TIF)

S1 Table LFQ dataset for CD81 co-IPs from Lunet N cells. Protein abundances in human hepatoma cell CD81 co-IPs. Listed are all detected proteins and the intensity differences of CD81-specific and control co-IPs for the experimental condition. Please refer to Excel file.
(XLSX)

S2 Table LFQ statistics for CD81 co-IPs from Lunet N cells. Welch test differences and p values for significant CD81 co-purifying proteins in human hepatoma cells. Tab 1: FDR = 0.01, s0 = 2; Tab 2: FDR = 0.004, s0 = 2. Please refer to Excel file.
(XLSX)

S3 Table Primary human hepatocyte LFQ dataset. Protein abundances in primary human hepatocyte CD81 co-IPs. Listed are all detected proteins and the intensity differences of CD81-specific and control co-IPs for cell from donor 1 and donor 2. Please refer to Excel file.
(XLSX)

S4 Table Protein expression in Lunet N human hepatoma cells. Protein abundances in whole cell lysates from human hepatoma cells. Listed are all detected proteins and their abundance as log_2_(iBAC) values. Please refer to Excel file.
(XLSX)
