# Supplementary figures and images for: Hepatitis C virus enters liver cells using the CD81 receptor complex proteins calpain-5 and CBLB
Source: PLoS Pathog. 2018 Jul 19;14(7):e1007111. doi: 10.1371/journal.ppat.1007111 (PMC6053247; doi:10.1371/journal.ppat.1007111)

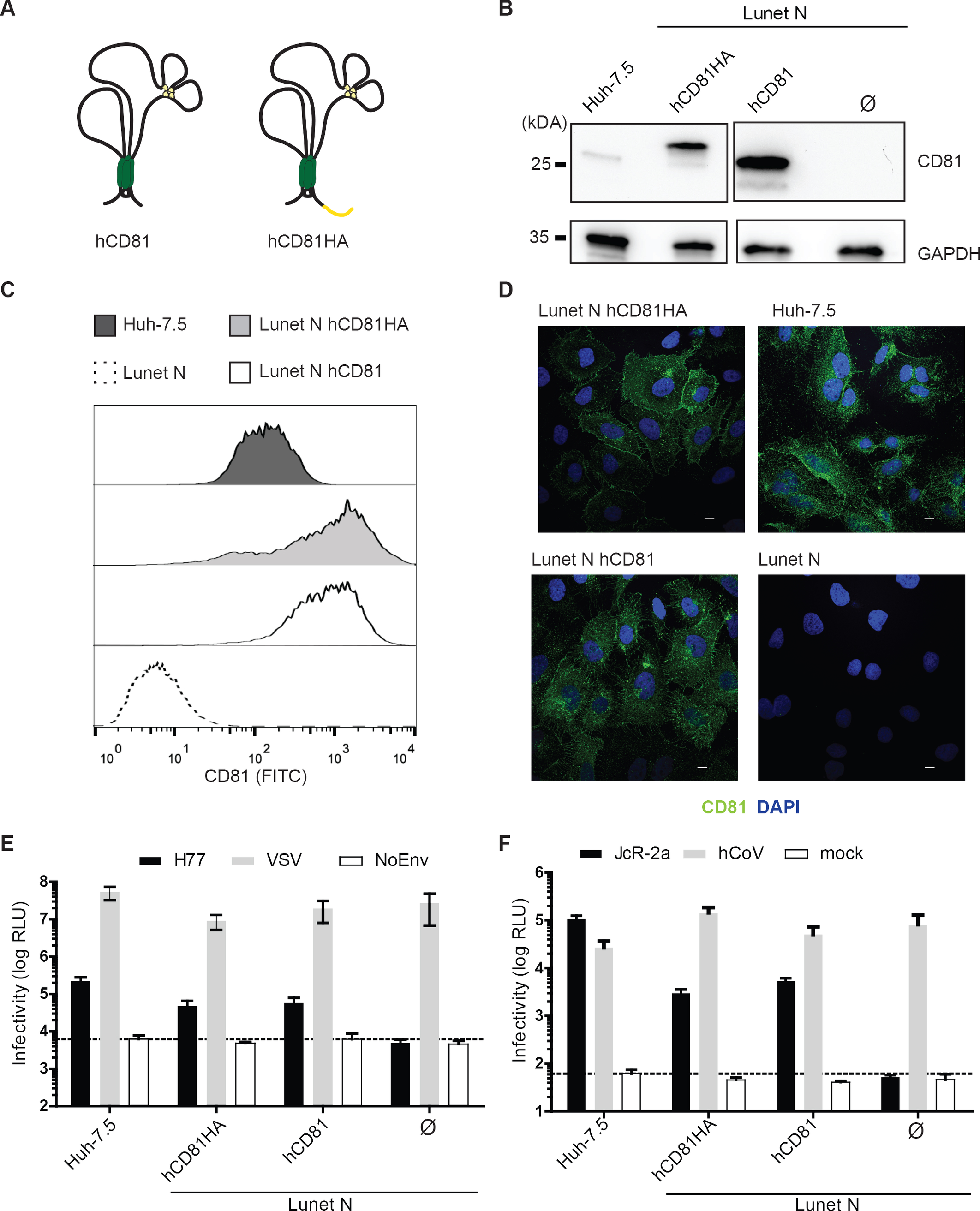

Supplement: S1 Fig — (A) Schematic representation of CD81-variants used in this study to analyze the CD81-interactome in human hepatoma cells. (B) Immunoblot analysis of CD81-expression in Lunet N derived cell lines used in this study. Protein expression was analyzed with an anti-CD81 antibody. Parental Huh-7.5 cells served as positive control, Lunet N cells served as negative control. GAPDH served as loading control. (C) Flow cytometric staining of surface-expressed CD81 in Lunet N derived cell lines used in this study. Parental Huh-7.5 cells served as positive control, Lunet N cells served as negative control. (D) Immunofluorescence staining of CD81 in Lunet N derived cell lines used in this study and after fixation and permeabilization. Parental Huh-7.5 cells served as positive control, Lunet N cells served as negative control. Stainings were analyzed by confocal microscopy. Scale bar: 10 μm. (E) Entry of lentiviral particles pseudotyped with glycoproteins from HCV GT1a (strain H77). Lentiviral particles pseudotyped with the VSV envelope proteins or with no envelope proteins served as positive and negative control, respectively. Infectivity was analyzed 72 h post infection by luciferase measurement. (F) Infection with the HCV reporter virus JCR-2a or a Coronavirus (CoV). Infectivity was analyzed 72 h or 24 h post infection, respectively, by luciferase measurement. Data from three independent experiments shown as mean +/- SEM. (TIF) [file ppat.1007111.s001.tif]

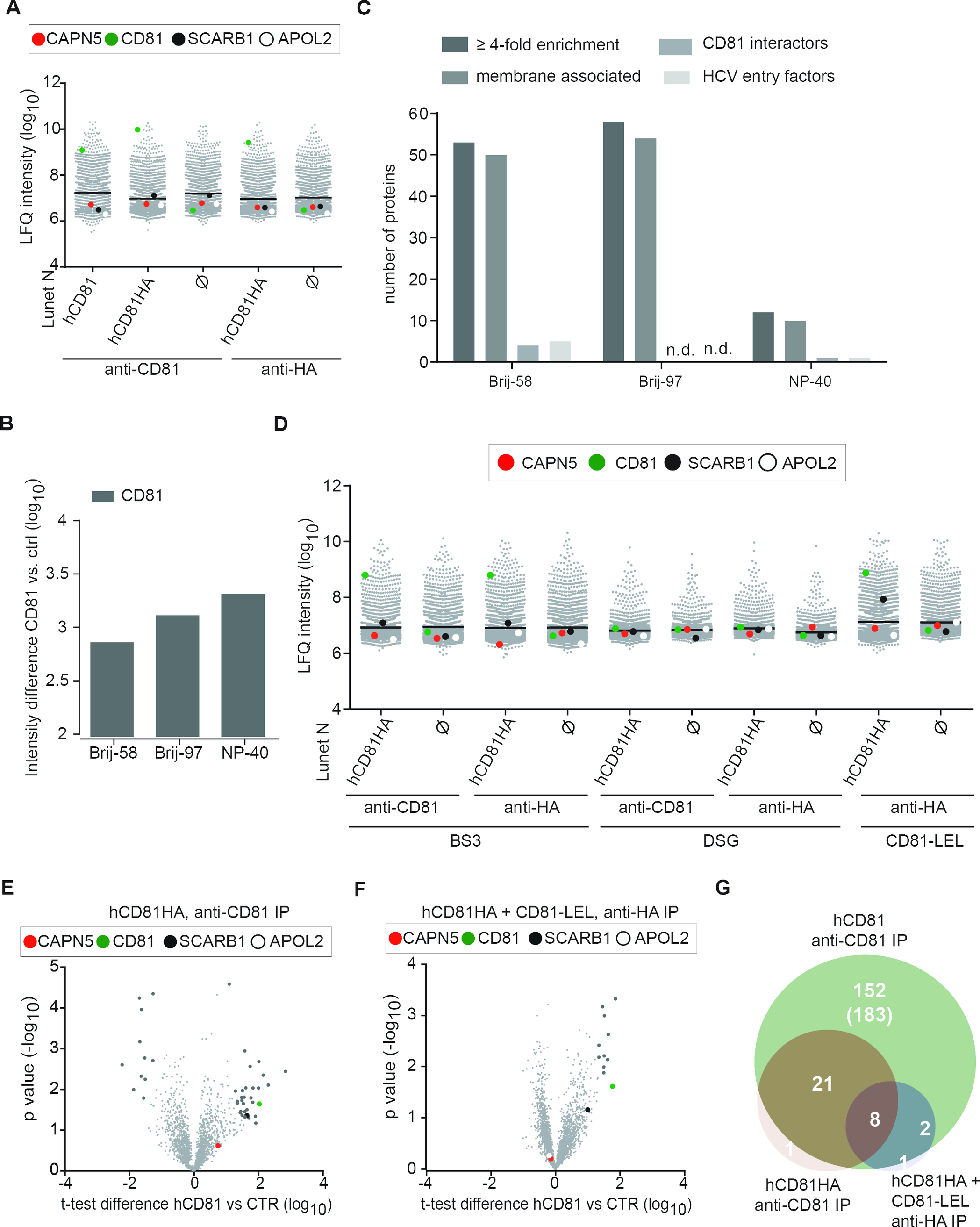

Supplement: S2 Fig — (A) Dot plot showing LFQ intensities of proteins in CD81- and HA-IPs from the indicated cell line lysed with NP-40 containing buffer. CD81 (green) and SCARB1 (black) served as positive and APOL2 (white) as negative control. Median of 4 biological replicates. (B) Pretest for choice of detergents: Intensity differences of CD81 in CD81-IPs from Lunet N hCD81 compared to Lunet N. Cells were lysed with buffers containing Brij-58, Brij-97 or NP-40 prior to IP. Mean of four biological replicates. (C) Pretest for choice of detergents: Number of proteins found to be ≥ 4-fold enriched in CD81-IPs from Lunet N hCD81 compared to CD81-IPs from Lunet N lysed with the indicated detergents. Among these proteins, the number of membrane associated proteins, known CD81-interactors and HCV entry co-factors are plotted. Mean of four biological replicates. n.d. = not detected. (D) Dot plot showing LFQ intensities of proteins in CD81- and HA-IPs from the indicated experimental conditions. CD81 (green) and SCARB1 (black) served as positive and APOL2 (white) as negative control. Shown are median logarithmic protein intensities of 4 biological replicates in co-IPs from cells after incubation with indicated cross-linkers or CD81-LEL prior to lysis with Brij-58 containing buffer. (E) Volcano plot visualizing two-sample t-test comparing LFQ intensities of proteins found in CD81-IPs from Lunet N hCD81HA and Lunet N. For each protein the t-test difference (log10) of CD81 versus control co-IP of 4 biological replicates is plotted against the p value (-log10). FDR = 0.01; s0 = 2. Proteins significantly enriched are highlighted in dark grey. CD81 (green), SCARB1 (black) APOL2 (white) and CAPN5 (red) are highlighted. (F) Volcano plot visualizing two-sample t-test comparing LFQ intensities of proteins found in HA-IPs from Lunet N hCD81HA and Lunet N cells incubated with soluble CD81-LEL. For each protein the t-test difference (log10) of HA versus control co-IP of 4 biological replicates is plotted aga [file ppat.1007111.s002.tif]

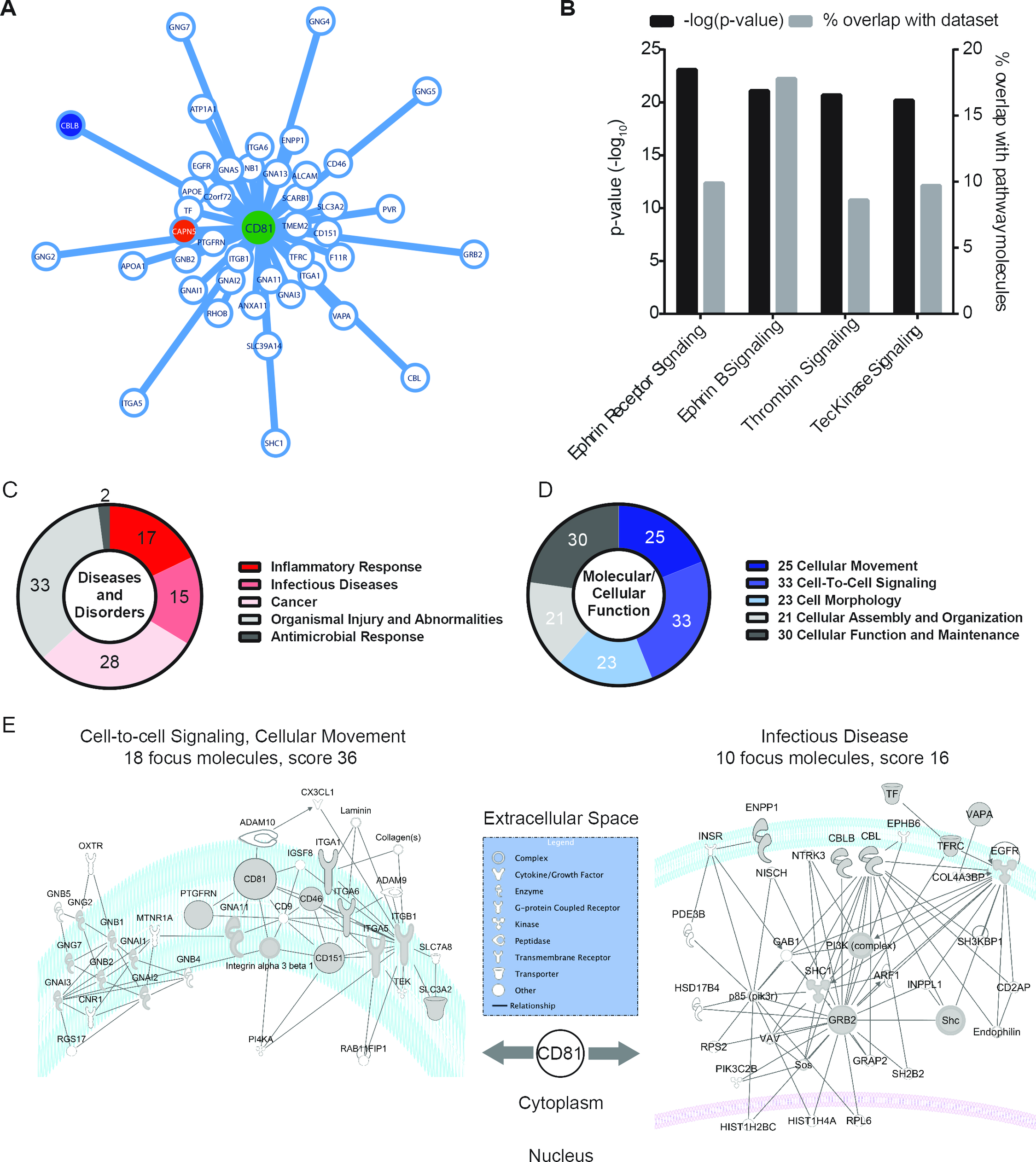

Supplement: S3 Fig — (A) Centered network depicting the 42 CD81 associated proteins identified in this study. Each node represents one protein and the length of the edges reflects the median enrichment score for each protein in CD81 co-IPs from hepatoma cells and primary hepatocytes. The nine in silico predicted interaction partners were assigned an artificial score and are depicted in the periphery of the network. CD81 (green), CAPN5 (red) and CBLB (blue) are highlighted. (B) Ingenuity pathway analysis of 42 CD81 interaction partners. P value (left y-axis) and overlap with in total reported pathway molecules (right y-axis) shown. (C, D) Ingenuity pathway enrichment analysis for diseases and disorders as well as for molecular and cellular function. The number in the donut chart represent the number of proteins. (E) Top two Ingenuity pathway molecular networks for the 42 liver cell CD81 interaction partners. Proteins identified in this study highlighted in grey. Additional network nodes shown in white. All proteins placed in their approximate subcellular localization. (TIF) [file ppat.1007111.s003.tif]

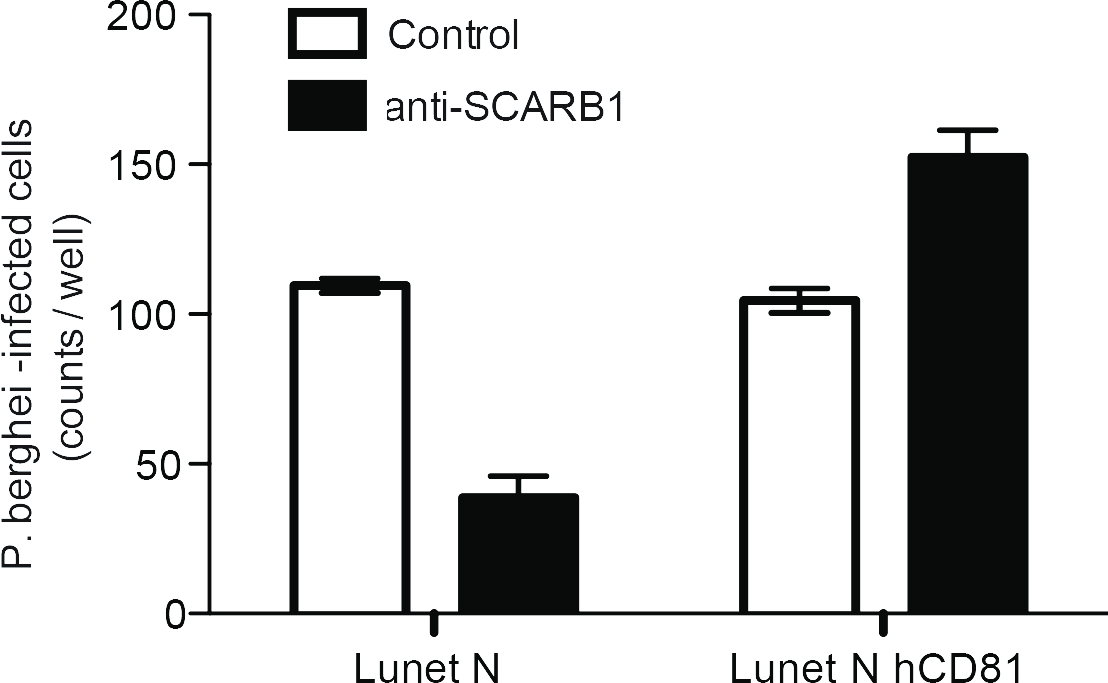

Supplement: S4 Fig — Lunet N and Lunet N hCD81 human hepatoma cells were infected with sporozoites of a P. berghei GFP reporter strain for 180 min in the presence or absence of an anti-SCARB1 antibody, then washed and further cultured until fixation at 36 hpi. Exoerythrocytic forms were quantified by fluorescence microscopy. Mean and SEM of 4 biological replicates shown. (TIF) [file ppat.1007111.s004.tif]

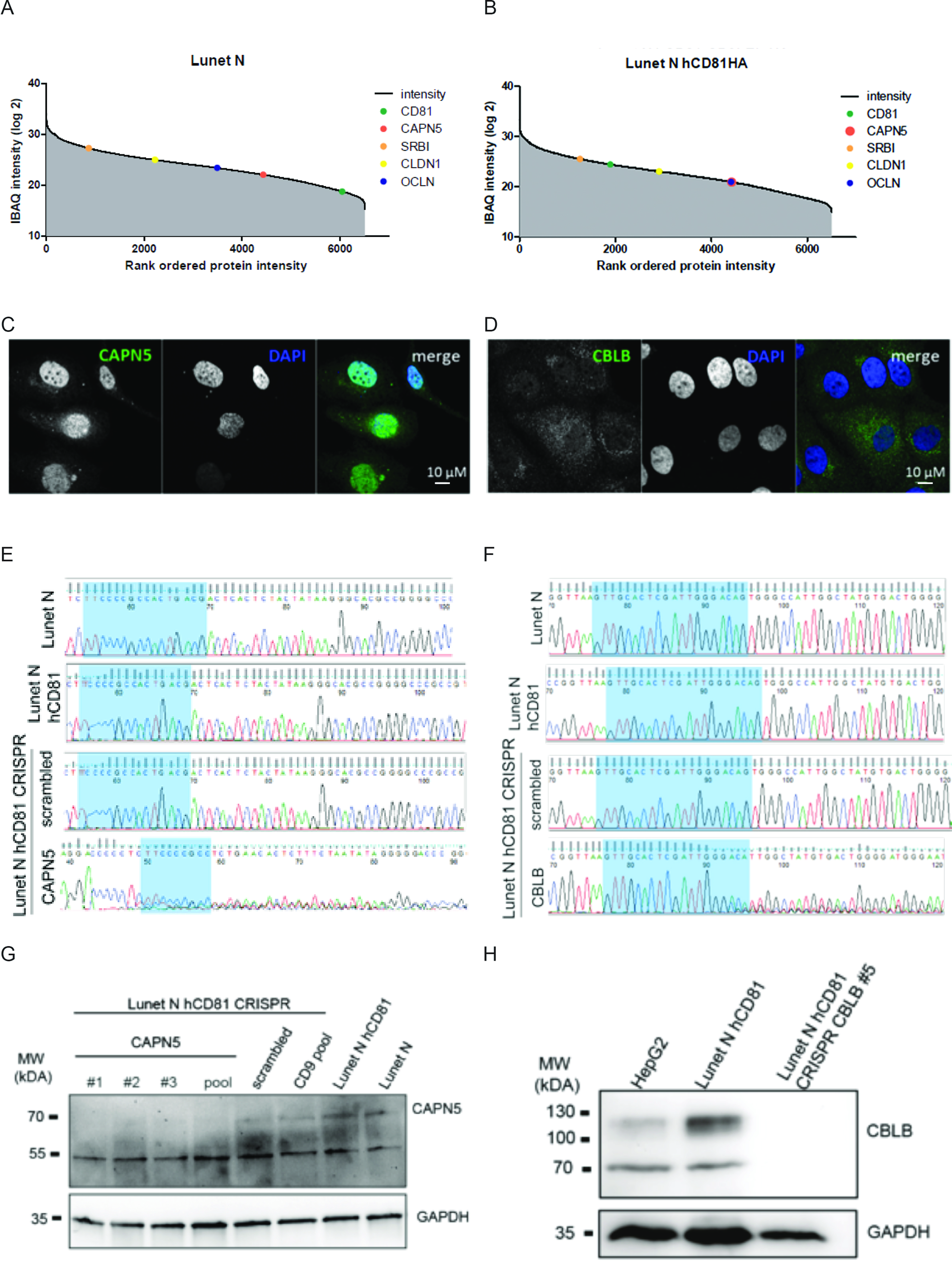

Supplement: S5 Fig — (A, B) Whole cell proteome quantification for (A) Lunet N and (B) Lunet N hCD81-HA cells. Expression level as iBAQ intensity highlighted for the CD81 interactor CAPN5 and the HCV entry factors CD81, SCARB1, CLDN1 and OCLN. (C, D) Immunofluorescence staining of (C) CAPN5 and (D) CBLB in Lunet N hCD81 after fixation and permeabilization. Stainings were analyzed by confocal microscopy. Scale bar: 10 μm. (E, F) Sequencing of genomic DNA isolated from parental and KO cell lines. The (E) CAPN5 and (F) CBLB genes were amplified and sequenced in the indicated KO and control cell lines. The sgRNA binding sites are highlighted in blue. A frameshift in the CAPN5- and CBLB-genes is visible in the respective KO cells, while the genes are intact in all other cell lines. (G, H) Immunoblot analysis of (G) CAPN5- and (H) CBLB-expression in parental and KO cell lines. HepG2 cells served as positive control for CBLB-expression, GAPDH served as loading control. (TIF) [file ppat.1007111.s005.tif]

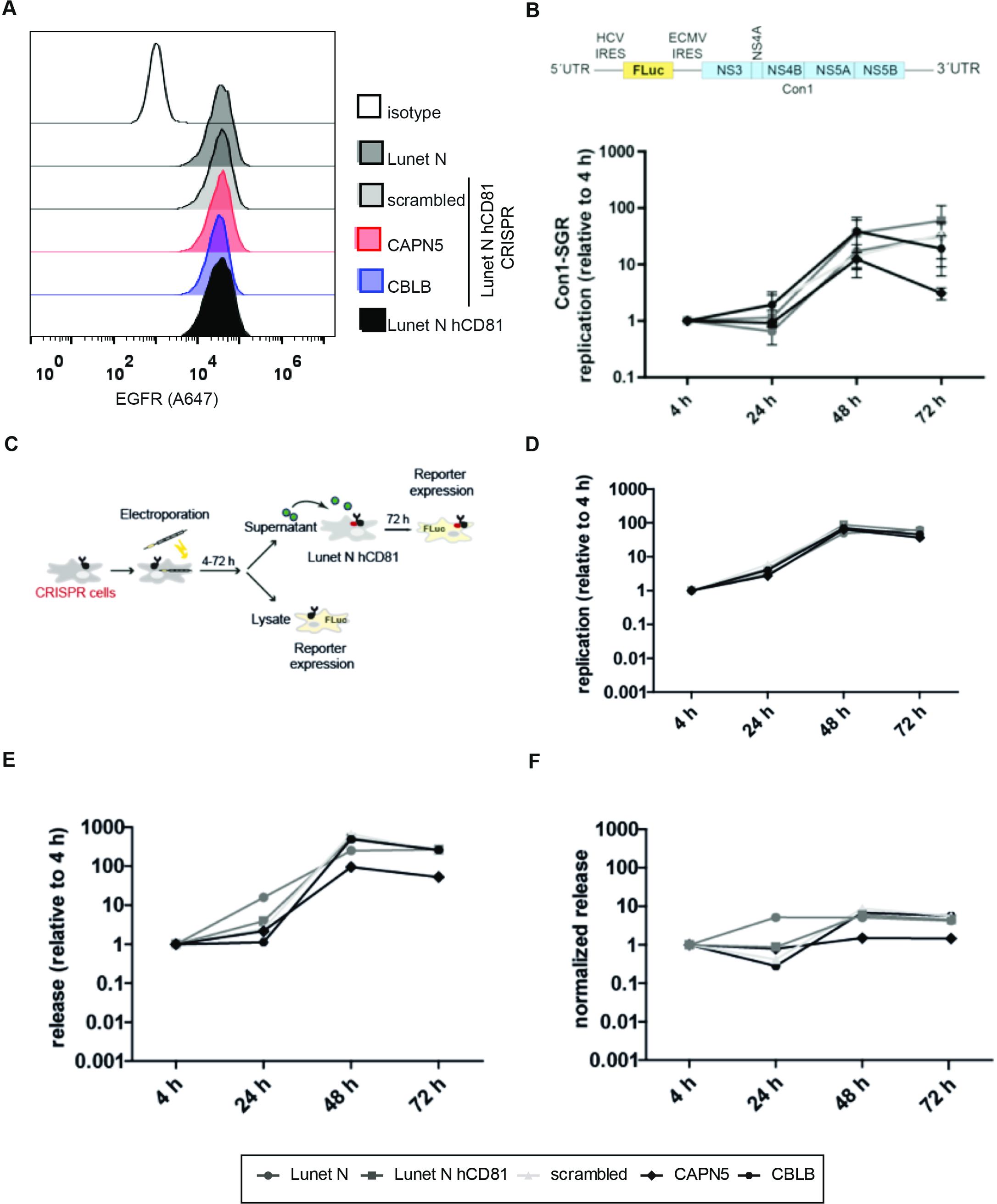

Supplement: S6 Fig — (A) Flow cytometric surface staining of EGFR in cells knocked out for CAPN5 (red) or CBLB (blue). Parental cells (black) served as positive control. Isotype control stainings or stainings with secondary antibody only (white) as negative controls. (B) Schematic overview of the HCV subgenomic replicon encoding a firefly luciferase and the NS proteins from HCV GT 1b isolate Con1, which was used to electroporate the parental and KO cell lines. Replication was monitored at the indicated time point post electroporation by luciferase measurement. Results are normalized to the 4 h time point to account for electroporation efficiency. Data from at least three independent experiments shown as mean +/- SEM. (C) Schematic overview of the experimental setup used to transfect parental and KO cell lines with a HCV reporter virus (JCR-2a) and analyze replication, assembly and release of new virus particles, as shown in D-F. (D) Replication was analyzed in the cell lysates by measuring luciferase activity at 4–72 h post electroporation. (E) Assembly and release was analyzed by transferring supernatant of electroporated cells to naïve Lunet N hCD81 cells. Infectivity was determined by measuring luciferase activity in the target cell lysates at 4–72 h post infection. (F) Infectious particle release was normalized to viral genome replication. Data from one representative experiment (D-F). (TIF) [file ppat.1007111.s006.tif]

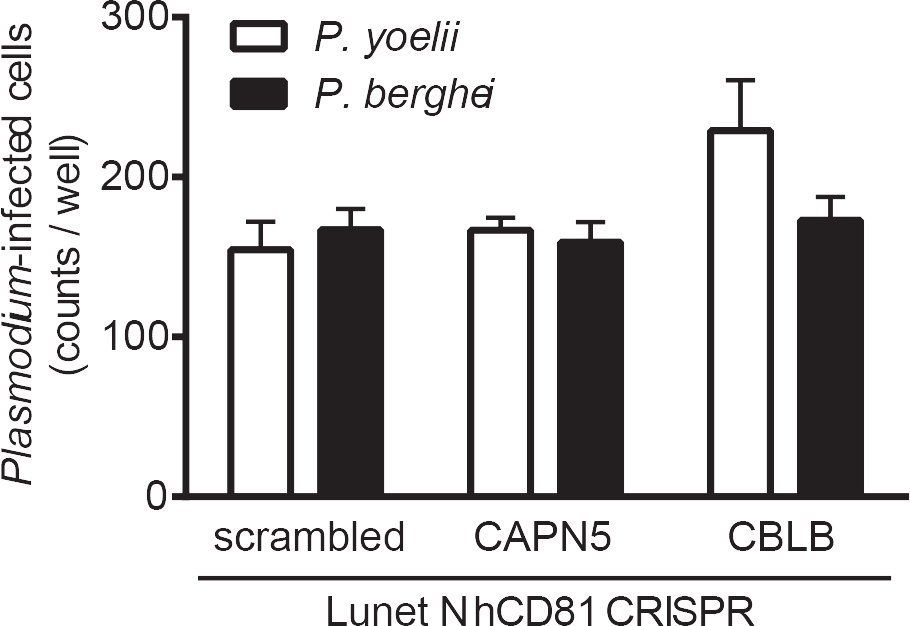

Supplement: S7 Fig — Lunet N hCD81 human hepatoma cells carrying the indicated sgRNA were infected with sporozoites of P. berghei or P.yoelii GFP reporter strains for 180 min, then washed and further cultured until fixation at 36 hpi. Exoerythrocytic forms were quantified by fluorescence microscopy. Mean and SEM of 5 technical replicates shown. Experiment performed twice for P. yoelii and once for P. berghei. (TIF) [file ppat.1007111.s007.tif]

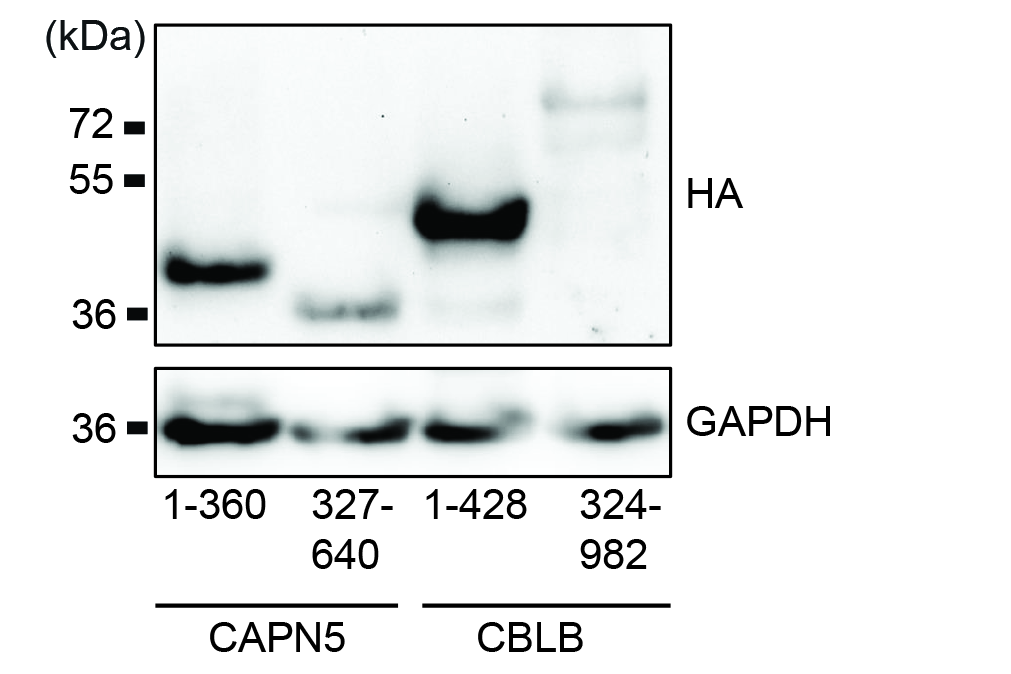

Supplement: S8 Fig — Cell lysates of Lunet N hCD81 human hepatoma cells expressing the indicated domain mutant of CAPN5 and CBLB fused to a tandem HA tag were analyzed by immunoblot against the C-terminal HA-tag. GAPDH served as loading control. (TIF) [file ppat.1007111.s008.tif]

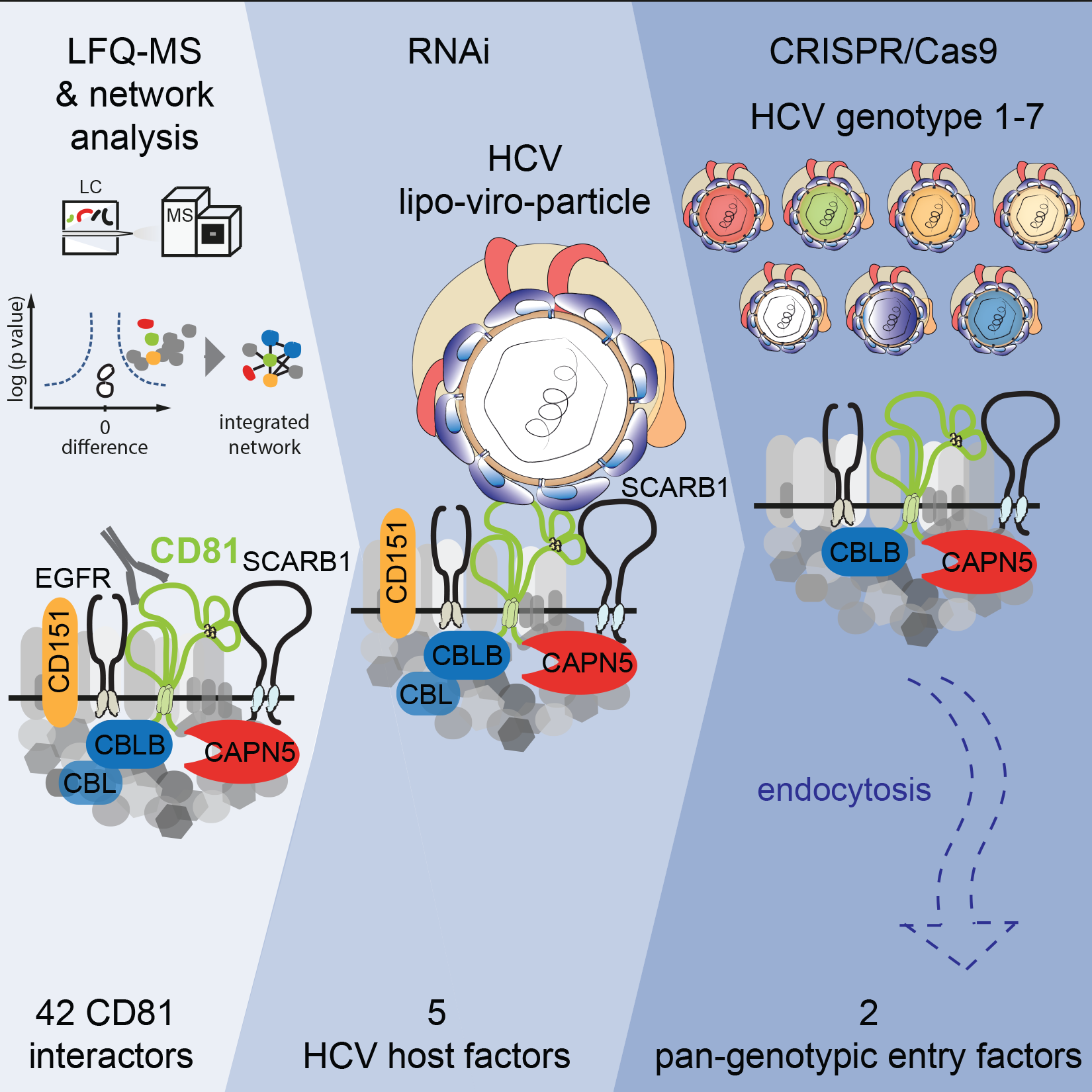

Supplement: S9 Fig — A combined label free quantification (LFQ)–RNA interference (RNAi)–CRISPR/Cas9 workflow identifies calpain-5 (CAPN5) and Casitas B-lineage lymphoma proto-oncogene B (CBLB) as hepatitis C virus (HCV) entry factors. CAPN5 and CBLB are critical host factors for all HCV genotypes and guide virus endocytosis and endosomal trafficking. (TIF) [file ppat.1007111.s009.tif]
